# Current State of Knowledge on Primary Sjögren’s Syndrome, an Autoimmune Exocrinopathy

**DOI:** 10.3390/jcm9072299

**Published:** 2020-07-20

**Authors:** Dorian Parisis, Clara Chivasso, Jason Perret, Muhammad Shahnawaz Soyfoo, Christine Delporte

**Affiliations:** 1 Laboratory of Pathophysiological and Nutritional Biochemistry, Université Libre de Bruxelles, 1070 Brussels, Belgium; dorian.parisis@ulb.be (D.P.); clara.chivasso@ulb.ac.be (C.C.); jason.perret@ulb.be (J.P.); 2 Department of Rheumatology, Erasme Hospital, Université Libre de Bruxelles, 1070 Brussels, Belgium; msoyfoo@ulb.ac.be

**Keywords:** Sjögren’s syndrome, autoimmune disease, physiopathology, treatment, diagnosis, review

## Abstract

Primary Sjögren’s syndrome (pSS) is a chronic systemic autoimmune rheumatic disease characterized by lymphoplasmacytic infiltration of the salivary and lacrimal glands, whereby sicca syndrome and/or systemic manifestations are the clinical hallmarks, associated with a particular autoantibody profile. pSS is the most frequent connective tissue disease after rheumatoid arthritis, affecting 0.3–3% of the population. Women are more prone to develop pSS than men, with a sex ratio of 9:1. Considered in the past as innocent collateral passive victims of autoimmunity, the epithelial cells of the salivary glands are now known to play an active role in the pathogenesis of the disease. The aetiology of the “autoimmune epithelitis” still remains unknown, but certainly involves genetic, environmental and hormonal factors. Later during the disease evolution, the subsequent chronic activation of B cells can lead to the development of systemic manifestations or non-Hodgkin’s lymphoma. The aim of the present comprehensive review is to provide the current state of knowledge on pSS. The review addresses the clinical manifestations and complications of the disease, the diagnostic workup, the pathogenic mechanisms and the therapeutic approaches.

## 1. Introduction

Sjögren’s syndrome (SS) is a chronic systemic rheumatic disease characterized by lymphoplasmacytic infiltration of the exocrine glands—especially salivary and lachrymal glands—responsible for sicca syndrome and systemic manifestations. The dreaded complication of this dysregulated and unabated lymphocytic activation is the development of lymphoma. SS can be “primary” if it occurs alone (pSS) or “secondary” (sSS) when it is associated with another autoimmune disease [1].

First medical descriptions of SS date back to 1882 when the German Theodor Karl Gustav von Leber (1840–1917) described for the first time a dry inflammation of the ocular surface under the name of “*keratitis filamentosa*”. Ten years later, the Polish surgeon Jan Mikulicz-Radecki described the case of a man with swelling of the salivary and lacrimal glands, a clinical picture still called Mikulicz syndrome today. At the same time, several cases of patients with ocular and oral dryness were described, whether or not associated with the existence of rheumatism or gout. Dr. W. B. Hadden (1856–1893) described the improvement of xerostomia in one of these patients with the use of an alkaloid called pilocarpine [2]. Despite the involvement of these physicians in the first medical descriptions of SS, only two famous names have remained attached to the disease: Gougerot and Sjögren. Henri Gougerot (1881–1955) was a French dermatologist who described in 1925 three clinical cases characterized by generalized mucous dryness (eyes, mouth, nose, trachea and vagina) associated with atrophy of the salivary glands (SG). He was the first to describe that xerostomia and ocular dryness are part of a larger sicca syndrome resulting from dysfunction of the exocrine glands or their autonomic innervation. In France, the term “Gougerot(-Sjögren) syndrome” is often used to describe pSS. Henrik Samuel Conrad Sjögren (1899–1986) was a Swedish ophthalmologist who was mainly interested in the dryness of the ocular surface. With his wife, Maria Hellgren, daughter of a well-known oculist, he described keratoconjunctivitis sicca (KCS)—distinct from vitamin A deficiency xerophthalmia—using Rose Bengal and methylene blue staining techniques. In 1933, in his PhD thesis, he described the cases of 19 women with KCS and 13 of whom had arthritis. He was therefore, the first to link KCS to a systemic disease beyond the field of ophthalmology. Unfortunately, his thesis was not successful, and he stopped his academic career but not his medical and scientific one. It was only in the years 1935–1943 that Sjögren’s work was recognized and that the term “Sjögren’s syndrome” has been used since. Finally, the autoimmune origin was recognized only in early 1960s [2]. Sjögren was awarded the title of “Doctor” in 1957 by the University of Gothenburg and the honorary title of “Professor” in 1961 by the Swedish Government. Henrik Sjögren died of pneumonia on 17 September 1986, several years after a disabling stroke [3,4,5].

## 2. Epidemiology

### 2.1. Prevalence

pSS affects 0.1% to 4.8% of the population with a female to male ratio of 9:1, depending on the cohort studied, classification criteria and methodology used [6,7]. Although pSS is considered a common disorder, its prevalence seems to be overestimated in some studies. Overall, 0.5–1% seems to be a commonly accepted estimate of the prevalence of pSS in the general population [7]. However according to a more recent meta-analysis of 7 studies, prevalence rate is 0.043% with a sex ratio of 10.72. The prevalence of pSS in Europe is higher than in Asia, 0.7122% and 0.045%. Sex ratio does not differ according to the geographic/ethnic origin of the populations studied [8].

### 2.2. Incidence

There is an overt heterogeneity of SS incidence among several studies. A meta-analysis reported an incidence rate of 6.92 per 100,000 person–years, with an overall average age of 56.2 years at diagnosis and an incidence rate ratio between women and men estimated at 9.29. Six Asian studies reported a relatively higher incidence ratio around 6 per 100,000 person–years. Both Slovenian and American studies reported an incidence ratio of 3.9 per 100,000 person–years. Finally, a Greek study estimated an incidence ratio between the two at 5.3 per 100,000 person–years. Data regarding the incidence of pSS in Africa, Oceania and South America are lacking [8].

## 3. Physiopathology of Sjögren’s Syndrome

**Figure 1:**
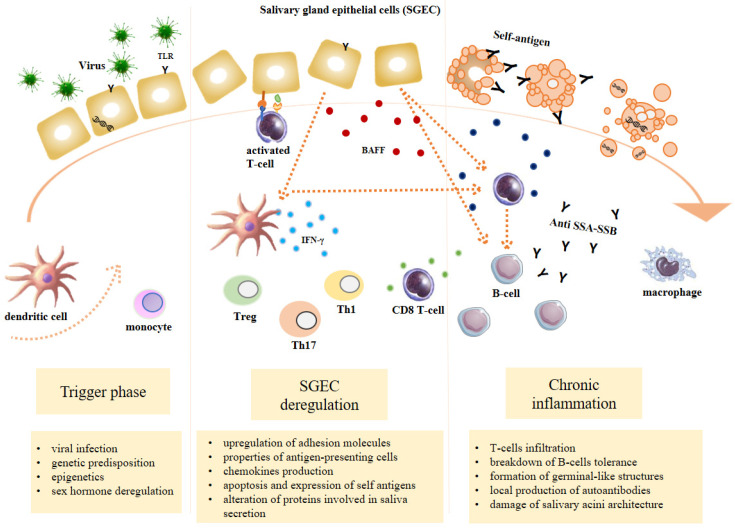
Overview of physiopathological mechanism underlying Sjögren’s syndrome (SS). Environmental triggers, such as viral infections, genetic predispositions, epigenetics and sex hormone deregulation, cause the disruption of salivary gland epithelial cell (SGEC), the production of type I interferon (IFN) and other cytokines such as B cell Activating Factor of the tumour necrosis factor (TNF) Family (BAFF) [11] and the alteration of proteins involved in saliva secretion. Dendritic cells, as well as SGEC acquire the characteristics of antigen-presenting cells capable of processing viral and self-antigens, leading to the activation of autoreactive T and B cells. Autoreactive T cells induce tissue damage through the release of cytotoxic granules and cause the exposure of autoantigens on the surface of SGEC. In addition, activated B cells produce autoantibodies that induce SGEC apoptosis and create an inflammatory microenvironment. This complex mechanism triggers a self-perpetuating cycle of autoimmunity.

SS is considered as a multifactorial process originating from the interaction between genetic factors and exogenous and endogenous agents able to trigger an abnormal autoimmune response mediated in particular by T and B lymphocytes [9]. The inflammation sustains, perpetuates and amplifies tissue damage and leads to a progressive functional impairment of the affected organs and a chronic inflammatory environment. Three recurrent events are generally associated with SS: (1) a trigger phase induced by environmental factors under specific epigenetic factors, genetic predisposition and hormonal regulation; (2) the dysregulation of normal salivary gland epithelial cell (SGEC) function; (3) a chronic inflammation characterized by SG infiltration made of lymphocytic cells, lymphocytes B hyperactivity and autoantibodies production [10] (Figure 1).

### 3.1. Trigger Phase

**Figure 2:**
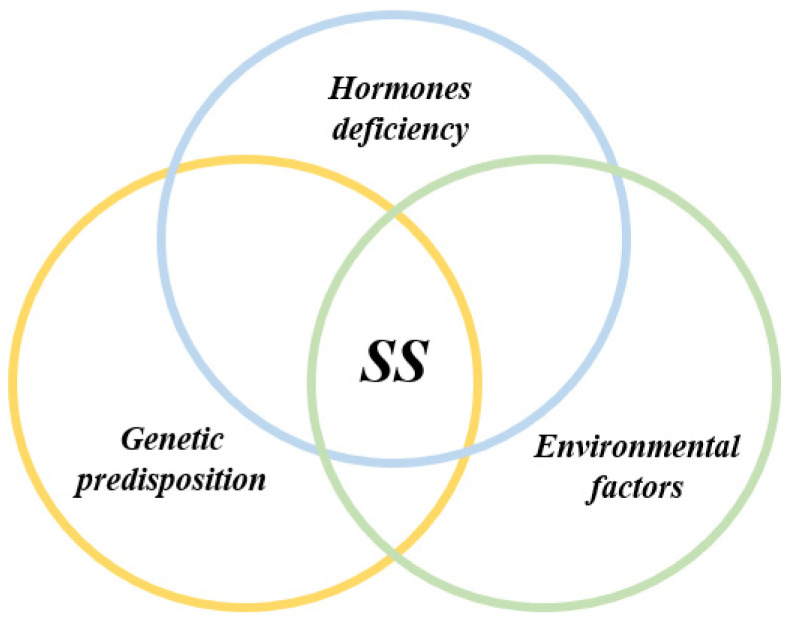
Factors involved in SS trigger phase.

In SS pathogenesis, a trigger phase is induced by environmental factors such as viral infections combined with genetic predisposition, epigenetic factors and sex hormonal regulation (Figure 2).

#### 3.1.1. Environmental Factors

According to the current physiopathogenic model of SS, environmental factors including viral infection lead to SGEC and Toll Like Receptors (TLRs) activation [12,13]. Primary viruses involved in SS induction include Epstein–Barr (EBV) viruses, Human T-lymphotropic virus type I (HTLVI), hepatitis virus C (HCV) and coxsackievirus [13].

EBV is a double stranded DNA virus appertaining to Herpesviridae family, with a strong tropism for B cells. EBV has often been associated with autoimmunity processes and diseases such as Rheumatoid Arthritis (RA), Systemic Lupus Erythematosus (SLE) and Multiple Sclerosis (MS) [14,15]. In addition, the high EBV load found in SG and lacrimal gland biopsies from SS patients as compared to controls [16,17] suggests its role in triggering the activation of the immune system. EBV is able to stimulate the production of proteins that mimic B cell receptor (BCR) and CD40 signalling and induce a strong B cell hyperactivity [18]. Recently, a correlation was established between past EBV infection and the presence of anti-Ro/SSA and anti-La/SSB autoantibodies in SS patients [19]. The RNA encoded by EBV binds TLR3 and induces the secretion of type I IFN and proinflammatory cytokines [20]. Another protein, the latent membrane protein 1 (LMP1) acting as a target for the EBV-induced cytotoxic T lymphocytes response may cause acini atrophy and SG lobule structure destruction observed in SS patients [21].

HTLV-1, a human endemic retrovirus in certain geographical areas such as Japan, has been reported to be present in SGEC [22]. In addition, epidemiologic studies revealed anti-HTLV-1 seropositivity in 23% of SS patients as compared to 3% in controls [23].

Coxsackie virus is a single stranded RNA virus belonging to the Picornaviridae family. A study has identified in SS patients a cross-reactivity between antibodies to the Ro60 epitope and 2B Coxsackie protein sharing 87% sequence homology [24]. However, these data remain controversial [25].

The role of HCV, a single stranded RNA small virus belonging to Flaviviridae family, has been examined in the initial triggering phase of SS. Clinical studies have shown that patients with HCV infection present sicca symptomatology, positive ocular tests, SG lymphocytic infiltration, and autoantibodies [26]. Therefore, HCV-associated SS (patients with HCV fulfilling SS 2002 classification criteria) is indistinguishable from pSS. On this basis, HCV chronic infection should be considered as an exclusion criterion for pSS as HCV infection could participate to SS development in a subset of patients.

Despite possible involvement of viral infection in SS, the most common antiviral drugs do not seem to show real benefit in the treatment of SS [26]. Indeed, as a viral infection may likely trigger onset of the disease, later antiviral treatment may manage a persistent infection but have no effect on the ongoing disease that may no longer be dependent on the presence of the initial viral infection.

#### 3.1.2. Genetic Predisposition

Genetic predisposition to SS plays a role in the trigger phase of the disease. A strong association between human leucocyte antigen (HLA)-DR and HLA-DQ alleles belonging to the group of major histocompatibility genes (MHC) class II genes and SS was observed throughout different populations including Caucasian, Japanese and Chinese populations [27]. All discovered haplotypes are in strong linkage disequilibrium, causing difficulties in establishing which of them contain the locus that confers the risk. SS patients with HLA-DQ1/HLA-DQ2 alleles display more severe autoimmune disease than patients with any other allelic combination at HLA-DQ [28]. In addition to the HLA system, most recent studies have focused their attention on polymorphic genes that code for molecules physiologically involved in apoptosis such as Fas and Fas ligand (FasL). Using MRL/lpr-murine model, a retrotransposon inserted in Fas gene was identified as playing a role in cell apoptosis and induction of progressive sialadenitis [29,30]. Fas/FasL gene polymorphisms have also been found in SS patients [31] but have not clearly been identified as disease-determining factors. Ro52 gene encoding the 52-kd Ro autoantigen display single nucleotide polymorphism (SNP) located 13bp upstream of exon 4 identified as significantly associated with the presence of anti-Ro 52kD autoantibodies in SS patients [32]. Numerous additional genes including IL-10 [33], TNF alpha [34], alpha chain of the IL-4 receptor [35], IRF5, STAT4 [36] and CXCL13 [37] also display a gene polymorphism possibly associated with SS as well. Recent studies carried out in several SS cohorts of different ethnicity have revealed additional candidate genes probably associated with the risk to develop the lymphoma in SS patients. The presence of a polymorphism in the tumour necrosis factor alpha induced protein 3 (TNFAIP3) gene is associated with the risk to develop the non-Hodkin’s lymphoma in a SS Caucasian cohort [38,39,40]. In addition, two polymorphisms of methylene-tetrapholate reductase (MTHFR) gene are considered risk factors for lymphoma in SS patients [41]. While gene polymorphism plays an indisputable role in the triggering phase of SS, the individual contribution of each genetic factor remains to be assessed [42].

#### 3.1.3. Epigenetic Factors

Several studies have analysed the contribution of epigenetics to SS and auto-antibodies production [43]. The epigenetic processes more closely linked to the disease are DNA methylation, miRNA, circular mRNA and long non-coding RNA function.

DNA methylation is a mechanism that consists in the addition of a methyl group from a methyl donor S-adenosylmethionine (SAM) to cytosine residues in the context of the CpG dinucleotide catalysed by DNA methyltransferases (DNMTs). In general, the addition of a methyl group onto DNA is associated with gene silencing due to a structural modification of chromatin. DNA methylation is one of most important mechanisms used by different type of cells to change their genetic expression such as the transition from naïve steady to effector B- and T-cells. An epigenome-wide analysis has identified several genes and epigenetic modification probably associated with SS [44]. The most frequent modification observed is the demethylation of several sites in SS patients’ genome. Labial SG DNA methylation is significantly reduced in SS patients as compared to the control subjects. This defect was conserved when the SGEC were primarily cultured. Apparently, the SGEC from SS patients were associated with a 7-fold decrease in DNMT1 and a 2-fold increase in demethylating partner Gadd45-alpha expression. This demethylation process was also associated in part with the infiltration of SG by B cells and the pathology severity [45]. Different studies have also reported a link between demethylating drugs and SS. In fact, mice receiving an oral administration of hydralazine or isoniazid (demethylating agents) for several weeks develop a pathology similar to SS in terms of immunological features and autoantibodies production. The signs of SS pathology disappeared after discontinuation of the drug [46]. A recent study conducted in CD19 + B cells and minor SG of SS patients has also identified a hypomethylation site on interferon (IFN)-regulated genes which induces an increase of IFN response activation normally observed in SS disease [47]. In addition, DNA demethylation of the pro-apoptotic death associated protein kinase (DAP-kinase) gene [48] and the runt-related transcription factor (RUNX1) gene in CD4 + T cells [49] have been associated with non-Hodgkin B cell lymphoma predisposition in SS. In conclusion, the genome methylation analysis represents a useful tool to identify links between epigenetic modifications in various cell types related to SS.

miRNAs are small endogenous non-coding RNAs that regulate gene-expression transcriptionally and post-transcriptionally. Interestingly, miR-17-92 cluster, is downregulated [50] and associated with a lymphoproliferative disease and autoimmunity [51,52] in SG of SS patients. Another study has shown increased levels of miR-146a that regulates the inflammatory response, inducing the repression of IRAK1 and the increase of TRAF6 expression which, in turn, promote NF-κB expression in the peripheral mononuclear cells of SS patients [53]. Aberrations in microRNA expression are often observed in various autoimmune diseases and for this reason they could be used as a potential diagnostic or prognostic biomarkers. Furthermore, the small size of mature miRNA offers a high level of stability that renders them useful in disease follow-up using paraffin embedded samples stored for long periods of time [54,55].

Circular RNA (circRNA) consist in a class of RNA generated after an alternative splicing process of pre-mRNA named “backsplicing”, in which a downstream 5′ donor links an upstream 3′ acceptor throughout a 3′ → 5′ phosphodiester bond. circRNa are divided in three subgroups: exonic circRNAs (ecircRNAs), intronic circRNAs (ciRNAs) and exon-intron circRNAs (EIciRNAs) [56]. Recent studies have observed that circRNA could be involved in development of autoimmune diseases such as RA, MS, SLE and SS [57]. A microarray analysis has identified 234 differentially expressed circRNAs between SS patients and healthy controls, whereby 2 are significantly upregulated and 3 downregulated in SS. Functional analysis has also shown that these circRNAs are related to arthritis and the presence of autoantibodies [58]. All this data taken into account, we can conclude that circRNAs could be used as biomarkers for a potentially valuable diagnostic tool for SS disease, but supplementary investigations assessing which of them is the most specific of pathology are necessary.

Long non-coding RNAs (lncRNA) are a novel class of functional non-translated RNAs with a length of over 200 nucleotides. Several studies revealed a strong link between lncRNAs and the immune responses [59]. The expression analysis of lncRNAs in SS patients has shown lncRNAs LINC00657, LINC00511 and CTD-2020K17.1 potentially associated with the disease. These 3 lncRNAs target different genes involved in B cell physiology and malignancy, including IL15, WDR5, GNAI2, LTßR, CBX8, BAK1, BAX ext [60]. IL15 and WDR5 play an important role in B cell proliferation and differentiation; GNAI2 regulates B cell trafficking to the lymph nodes [61]; LTßR and CBX8 are involved in GC formation in inflamed tissues [62,63], and BAK1 and BAX are overexpressed in B cell lymphoma [64]. These results illustrate an important role of lncRNAs in multiple processes and the understanding of their modulation and function could provide deeper insight into the pathogenesis of SS and facilitate the identification of novel therapeutic strategies.

#### 3.1.4. Sex Hormones Deregulation and X-Chromosome Linked Factors

Nine out of ten SS patients are women and generally during menopause [65]. The strong predisposition of women to develop SS clearly demonstrates the role of sex hormones as a risk factor of the disease. In a recent case-control study, pSS in women was associated with lower oestrogen exposure and lower cumulative menstrual cycling time compared to sicca controls. Conversely, an increasing oestrogen exposure was negatively associated with development of pSS [66]. Finally, an effect of X chromosome per se is also evoked since men with Klinefelter’s syndrome have a higher risk of developing pSS—20 times higher—compared to healthy men, despite normal sex hormone levels [67,68]. Similarly, the association between pSS and mixed connective tissue disease has been reported in a 16-year-old Japanese patient with trisomy X [69].

Androgens suppress the inflammation and enhance the function of lacrimal glands in female SS mouse models (MRL/MpJ-Tnfrsf6lpr[MRL/lpr]) [70]. The androgens could help maintaining acini structure in healthy SG, while their reduction observed in SS patients could cause a decrease in integrin expression and probably a dysregulation of acini architecture [71]. SS patients present low levels of androgen hormones both in the bloodstream and in SG [72]. In Klinefelter’s syndrome associated SS and SLE, correction of hypogonadism by testosterone therapy for 60 days leads to remission in one case-series report [73].

Healthy ovariectomized C57BL/6 mice display an exocrinopathy with autoimmune characteristics similar to SS including SG focal adenitis, lacrimal glands lesions, Ro/SSA, La/SSB and α-fodrin autoantibodies [74]. Similarly to ovariectomized mice, both mice rendered deficient in aromatase, an enzyme important in the biosynthesis of oestrogens, as well as mice that received an aromatase inhibitor develop a lymphoproliferative autoimmune disease resembling SS [75,76]. How oestrogen deficiency promotes autoimmune lesions remains unclear. However, one putative explanation could be that oestrogen deficiency stimulates SGEC to secrete IFN-α and IL-8, and to express MHC class II, enabling them to act as antigen-presenting cells. Oestrogen deficiency is responsible for RbAp48 overexpression, which induces p53-mediated apoptosis in exocrine glands [77]. In another study, transgenic mice overexpressing RbAp48 develop SS-like exocrinopathy characterized by an increased propensity to apoptosis and the acquisition of an active immunocompetent role by epithelial cells, producing IFN-γ and IL-18 [78]. In primary cultures of human SG cells, pre-treatment with 7β-estradiol impede IFNγ-induced upregulation of ICAM-1 in control group but not in pSS group. These data suggest a protective role of oestrogens on epithelial activation and the existence of a deficient estrogenic responsiveness in pSS [79]. Not surprisingly, the use of aromatase inhibitors in the treatment of breast cancer is associated with arthralgia or even authentic SS [80,81,82].

**Figure 3:**
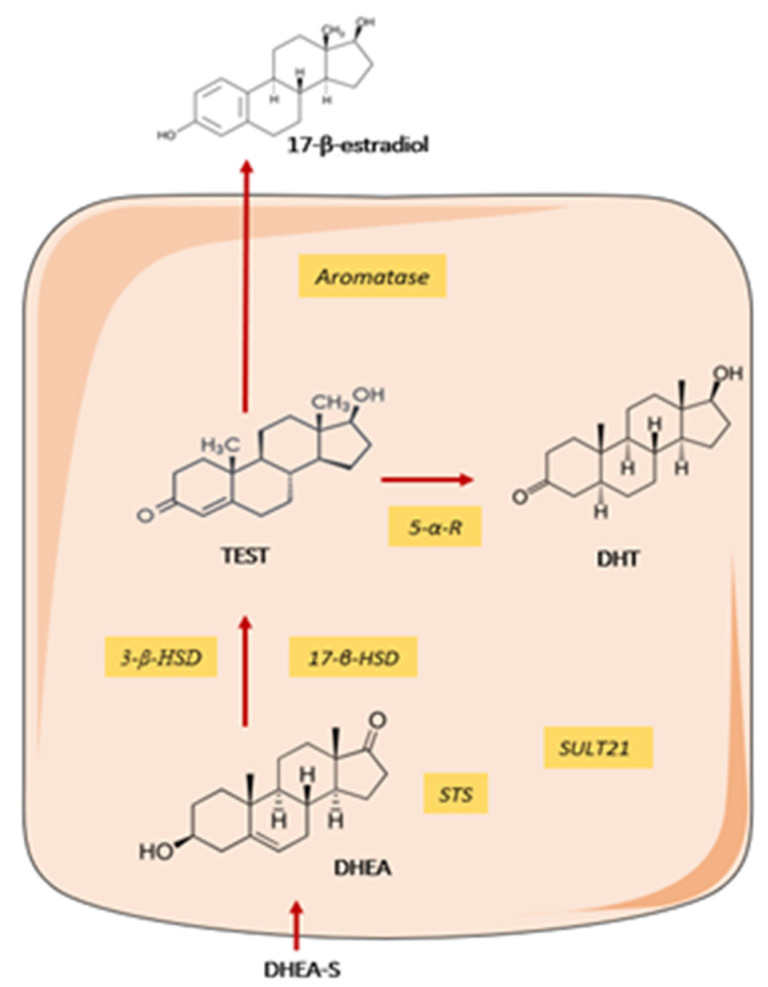
Intracrine steroidogenic machinery in healthy acinar cells. The figure shows the conversion of dehydroepiandrosterone (DHEA) to active sex steroids. STS: steroid sulphatase, SULT2B1: sulfotransferase 2B1, HSD: hydroxy steroid dehydrogenase, 5-α-R: 5α-reductase, TEST: testosterone, DHT: dihydrotestosterone. DHEA-S: DHEA-sulphate.

Humans and other primates, secrete large amount of sex steroid precursors, such as dehydroepiandrosterone (DHEA) and DHEA-sulphate precursors, metabolic intermediates in the biosynthesis of androgens and oestrogens. According to tissue needs, the prohormones are directly processed within tissues. DHEA is present in low concentrations in patients with SS as compared to age-matched healthy controls [83]. Several studies have shown that human MSG possess an organized intracrine machinery capable to convert DHEA(-sulphate) pro-hormone to its active metabolites, dihydrotestosterone (DHT) and 17β-oestradiol [84] (Figure 3). However, the non- functionality of this enzymatic machinery in MSG from SS patients could account for the diminished local concentrations of DHT and androgen-regulated biomarker Cysteine-Rich Secretory Protein 3 (CRISP-3) in SS patients [85].

Taken together, these data suggest that women affected by SS at menopause, when the levels of testosterone produced by the ovaries has already declined, may be particularly vulnerable to androgen deficiency because the only source of DHT in SG is dependent on local conversion of DHEA. Whereas in men, the level of systemic androgens produces by gonads may satisfy the specific needs of SG, not requiring the intermediate metabolite.

### 3.2. SGEC Deregulation

#### 3.2.1. Upregulation of Adhesion Molecules

According to recent observations, several SS pathogenic models could explain the role of SGEC in glandular damage. The current SS pathogenic model is the “autoimmune epithelitis”. This model considers SGEC as a crucial player in the initial triggering phase of the disease [86]. SGEC from SS patients express significantly higher levels of TLRs mRNA levels, including TLR-1, TLR-2, TLR-3 and TLR-4 as compared to control SGEC [87]. Under physiological conditions, TLRs are activated by the recognition of pathogen-associated molecular patterns (PAMPs) derived from microorganisms and endogenous mediators of inflammation known as danger-associated molecular patterns (DAMPs) [88]. TLR signalling pathway acts as link between innate and adaptive immunity in autoimmune diseases. Indeed, upon activation, TLRs recruit adapter proteins in order to propagate the intracellular signal that results in the transcription of genes involved in inflammation, immune regulation, cell survival and proliferation and subsequent activation of the immune system. TLR signalling in SGEC upregulates several molecules such as MHC class I and class II, costimulatory molecules such as B7.1 (CD80) and B7.2 (CD86) and adhesion molecules 1 (ICAM-1) [89].

#### 3.2.2. Antigen-Presenting Cell Properties

The expression of MHC class I, MHC class II, costimulatory molecules and adhesion molecules on SGECs empower them to present antigen to T cells (acting as non-professional antigen presenting cells).

#### 3.2.3. Chemokines Production

The activation of Interferon Regulatory Factor (IRF) and nuclear factor kappa-light-chain-enhancer of activated B cells (NFkB) pathways increases the production of inflammatory cytokines, including type I IFN, tumour necrosis factor-α (TNF-α), interleukin(IL)-1, IL-6 and BAFF [90].

#### 3.2.4. Apoptosis and Expression of Self-Antigens

In addition to chemokines production, the ribonucleoproteins, normally hidden from the immune system, are exposed on the cell surface. In particular, the expression of antigen Ro/SSA and La/SSB proteins on apoptotic SGEC promotes the initiation of autoimmunity.

#### 3.2.5. Alteration of Proteins Involved in Saliva Secretion

Apoptosis of the acinar epithelial cells and altered expression and distribution of proteins involved in saliva secretion has been proposed as possible mechanisms responsible for the impairment of secretory function of SS SG. For example, an increase in AQP3 expression was observed at the apical membrane of acinar cell of SG from SS [91], while AQP1 [92] and AQP4 [93] expression was decreased in myoepithelial cells. Rituximab treatment, used in SS patients to deplete B cells, increases AQP1 protein expression in myoepithelial cell and induces an improvement of saliva flow [94]. These data could suggest a crucial role to AQP1 in saliva secretion. However, AQP1-null mice model has shown that this protein is not essential for saliva production [95]. Nevertheless, one cannot exclude a compensatory effect in such mouse models, whereby other AQPs could be alternatively used. In contrast, AQP5 is today considered the most important protein involved in saliva secretion [96]. Under physiological conditions, AQP5 translocates from the intracellular vesicular compartments to the apical membrane of SG acinar cells after activation of muscarinic and adrenergic receptors [97]. In SS patients and SS mice models, aberrant localization of AQP5 has been observed [98], which is predominately basolateral instead of apical [99,100,101]. The reason why the AQP5 localization is altered is still unknown but several hypotheses have been proposed.

The presence of autoantibodies against M3 receptor could impair its activation and block the translocation signal normally sent to AQP5 [102]. Another possible mechanism could be the alteration of protein–protein interactions between AQP5 and its partner proteins [103]. Prolactin inducible protein (PIP) is a known AQP5 protein partner in lacrimal glands in mice models. Aberrant binding of PIP to the c-terminal domain of AQP5 impairs AQP5 trafficking to the apical membrane of epithelial cells [104]. Lastly, the inflammatory environment that characterizes SS disease could also directly or indirectly be involved in these modifications [105,106]. IFN-γ for example, contributes to SS pathogenesis inducing SG apoptosis and expression of several chemoattractant cytokines and enhancing the antigen presenting function of epithelial cells [107,108,109]. IFN-γ administration leads to increased production of anti-M3R antibody, which affect the SG secretory function in response to an adequate stimulus [110]. Neutralization of IFN-γ in anti-programmed death ligand 1 (PDL1)-treated non-obese diabetic (NOD)/ShiLtJ mice improves AQP5 expression and saliva secretion [111]. TNF-α is another pro-inflammatory cytokine that is increased in SS [112]. Elevated TNF-α levels in both serum and SG has been observed in SS patients compared to controls [113]. In human SG acinar cells, TNF-α treatment down-regulates the expression of AQP5 [114]. The injection of antibodies against TNF-α in NOD mice reduces SG inflammatory foci and increases AQP5 protein expression [115]. It seems clear that correct expression, trafficking and localization of AQP5 are essential to overcome the impaired salivary secretion process and the combination of inflammation, antibodies production, protein–protein interaction and salivary epithelial cells deregulation are probably involved in the hypofunction of SG of SS patients.

### 3.3. Chronic Inflammation

#### 3.3.1. T-Cell Infiltration

In the early stages of SS, the lymphocytic infiltrates, present in SG from SS patients, are constituted by a vast majority (>75%) of T lymphocytes being mostly CD4 T cells [116]. However, saliva from SS patients contains greater Th1 cytokines than saliva from controls [109,117], including IL-1β, IL-6, tumour necrosis factor (TNF)-α, and IFN-γ [118]. Th2-derived cytokines, such as IL-10 and IL-4, were also found in greater quantity in SG tissue from SS patients than in controls [119]. The two T cell responses are in a dynamic balance with a predominance of Th1 activity in patients suffering from SS [120]. In patients with SS, the activated T cells respond to an intense antigenic stimulus, such as the recognition of Ro and La autoantigens expressed on blebs of apoptotic cells [121], which induces a proliferative response [122]. Therefore, T-cell recognition of self-antigens and their subsequent activation are crucial for the cascade of events leading to the development of SS pathology. T cells may proliferate locally in SG or be re-directed by chemokines from the circulation to the glands. Two chemokines involved in the attraction of T-cells in SS SG are CXCL9 and CXCL10 [123]. In SS SG, T cells are likely to be involved in the disruption of the glandular architecture throughout the apoptosis mechanism mediated by FasL pathway [124], by a direct cytotoxic activity involving the release of perforin and/or secretion of cytokines and by the activation of B cells [125]. Th17 cells represent another subpopulation of T-cells strongly activated in SS patients [126]. In general, Th17 plays an important physiological role in mucosal defence in healthy individuals. In SS patients, the activated Th17 cells promote inflammation by secreting IL-6, IL-17, IL-21, IL-22 and IL-23 [127,128,129]. Follicular helper T cells have been shown to play an important role in lymphoid follicle formation and ectopic germinal centre formation in SS SG [130]. During pathology, SGEC induce activation and differentiation of T helper to T follicular helper by the release of IL6 and ICOS ligand expression. The activated follicular cells in turn secrete IL-21 cytokine which mediates B cell maturation and proliferation [131]. In conclusion, the combined activation of T-cell subtypes creates an optimal environment for detrimental B cell activation and the breakdown of tolerance.

#### 3.3.2. Breakdown of B Cells Tolerance

Under physiological condition, B cells originate in the bone marrow from haematopoietic stem cells and during their development undergo several stages of selection because of a large portion of self-reactive and polyreactive B cell are normally generated [132]. The first checkpoint removes the polyreactive B cells in the bone marrow (central tolerance checkpoint), the second in the periphery ensures that only a small amount of self-reactive, and polyreactive mature naïve B cells survive. Finally, a third tolerance checkpoint called pre-germinal centre checkpoint, excludes self-reactive naïve B cells from entering B cell follicles [133].

A recent study has revealed the existence of deficiencies in both early and late B cell tolerance checkpoints in patients with SS. Indeed, the accumulation of circulating autoreactive naïve B cells in SS suggests an impairment of the autoreactive B cell clearance during the early peripheral tolerance checkpoints and an increased frequency of autoreactive unswitched and switched memory B cells reveals a possible impairment also in pre- and/or post-germinal centre tolerance checkpoints [134]. These observations have also been made in patients with SLE, RA and type 1 diabetes [135,136]. B cell depletion using anti-CD20 antibodies in Id3 knockout mice model leads to a significant histological improvement associated with a recovery of saliva secretory function and corroborate the hypothesis that B cells could play an important role in SS disease [137].

B cell hyperactivity is an important hallmark of SS. Two cytokines have been shown to be fundamental in B cell survival and proliferation: B cell Activating Factor of the TNF Family (BAFF) and APRIL (A proliferating ligand) [138]. Once SG tissue infiltration is established, a large number of cells such as dendritic cells, monocytes and macrophages but also SGEC and T lymphocytes can secrete BAFF. BAFF overexpression has indeed been documented in SS as well as in other systemic autoimmune diseases and has been correlated with autoantibodies [139].

#### 3.3.3. Formation of Germinal-Like Structures

Germinal centres (GCs) were described for the first time by Walther Flemming in 1884 [140]. GCs are specific region in secondary lymphoid tissues such lymph nodes and spleen. GCs provide the environment for proliferation of mature B cells, differentiation and mutation of their immunoglobulin variable-region gene segments during a process called somatic hypermutation, which generates a diversity of clones. Following this process, the cells migrate from the dark zone to the lighter zone of the lymphoid tissues, where the affinity of immunoglobulins is tested on follicular dendritic cells (FDC) and follicular helper T cells (TFH) cells presenting the antigens. The non-selected cells undergo apoptosis while the selected cells are stimulated by T cells to undergo class switch recombination and differentiation into antibody-producing plasma cells or memory B cells [141,142]. SG from SS patients can contain similar GC structures made of T, B, and plasma cells, macrophages, and follicular dendritic cells [143]. Given the strong similarity of SG GC with the lymphoid organ GC, the SG GC observed in SS patients were defined as ectopic GC-like structures, also known as “tertiary lymphoid organs” [144]. Several studies have reported the association between GCs and the immunopathological features of SS [145]. Other important studies have observed a 6.5- to 15.6-fold increased risk to develop non-Hodgkin lymphomas in SS with an elevated presence of GCs [146,147].

#### 3.3.4. Local Production of Autoantibodies

The most common and studied antibodies in SS patients are those directed against the autoantigens Ro/SSA and La/SSB [148]. Anti-Ro, Anti-La, anti-SSA and anti-SSB were originally described as four antibodies directed against antigens expressed by salivary and lacrimal glands tissues from SS patients. Later, anti-Ro and anti-La were shown to be the same antibodies as anti-SSA and anti-SSB, respectively [149,150].

Ro antigen is constituted of two distinct Ro proteins of 52 and 60 kDa, with the latter binding to small cytoplasmic RNAs known as hY RNAs. The Ro52 protein, also known as TRIM21, is frequently targeted by SS antibodies, which makes it a useful diagnostic marker, but its function and why it becomes a target protein in a lot of rheumatic diseases is not completely understood. Ro52 is a member of the tripartite motif (TRIM) protein family, and it plays an important role in the ubiquitination of proteins. Several targets have been suggested as substrate of Ro52 activity, including various members of the IFN-regulatory factor (IRF) transcription factor family. The most speculated hypothesis attributes to Ro52 a role of IFN negative regulator. Indeed, in a Ro52-null mouse, the lack of ubiquitination mediated by Ro52 leads to an aberrant expression of type I IFNs and proinflammatory cytokines, such as IL-6, IL-12, IL-23, and TNF-α [151]. La/SSB antigen is a 48 kDa phosphorylated protein located in the nucleus and the cytoplasm. La/SSB binds to many RNA molecules newly synthesized by RNA polymerase III [152]. These two antibodies are detected in 50% to 70% of primary SS patients, but the anti-La/SSB alone is observed in only 2% of patients [153,154].

In most cases, anti-Ro/SSA and anti-La/SSB are correlated with severe dysfunction of the exocrine glands, associated with parotid gland enlargement and large number of lymphocytic infiltrates in the MSG [155,156].

Other antibodies believed to be pathogenic in SS are anti-centromere antibodies (ACA), anti-citrullinated protein antibodies (ACPA), anti-carbonic anhydrase II antibodies, anti-aquaporin-5, anti-muscarinic receptor 3 (anti-M3R) and anti-fodrin antibodies. ACA are directed against six antigens associated with the centromere (complex of kinetochore proteins). The incidence of ACA antibody ranges from 3.7% to 4% [157,158]. ACPA are directed against fibrin and fibrinogen, vimentine and alpha-enolase (CEP-1). In general, ACPA antibodies are the marker most observed in rheumatoid arthritis but are usually present in low concentrations in pSS as well, in about 3–22% of cases [159]. Anti-carbonic anhydrase II antibodies have been detected in 12.5–20.8% of SS patients and also play a pathogenic role in renal tubular acidosis (RTA) [160,161]. In fact, immunization of mice with human carbonic anhydrase II resulted in autoimmune sialadenitis, production of anti-carbonic-anhydrase-II antibodies and urinary acidification defect [162,163]. Anti-AQP5 antibodies were observed to be associated with serologic and histopathological features of SS [164]. Anti-M3R antibodies are present in serum of up to 90% of subjects with SS [165]. Antibodies against alpha-fodrin are detected in serum samples from patients with primary or secondary SS, especially in patients with sicca symptoms. However, anti-alpha-fodrin antibodies do not represent a sensitive nor a specific serological marker of SS [166]. Other novel tissue-specific autoantibodies are currently under investigation: autoantibodies against salivary protein 1 (SP-1), parotid secretory protein (PSP) and carbonic anhydrase 6 have been described in pSS and non-pSS patients with chronic pain, which may help to understand and diagnose early pSS and pSS-associated widespread pain syndrome in the future [167]. Anti-cofilin-1, anti-alpha-enolase and anti-RGI2 antibodies are associated with pSS MALT lymphoma [168]. Other autoantibodies have also been described to be more frequently found in pSS patients and variously associated with the clinical and biological characteristics of the disease [168]. Table 1 summarizes the novel autoantibodies that have been detected in pSS patients.

#### 3.3.5. Damage of Salivary Acini Architecture

One of the pathomorphological characteristics of SG from SS patients is the presence of focal infiltration made of lymphocytic cells. The focus infiltrate is defined as the “focus score” and “focus score = 1” is a group of 50 or more lymphocytes per 4 mm^2^ of tissue [196]. SG infiltration is normally associated with destruction and fragmentation of the glandular tissue, acinar hyperplasia and replacement of acinar cells with fatty or fibrotic infiltrations [197]. These events lead to a deep modification and impaired function of the glandular tissue. An architectural disorganization of the epithelial cells has been described in the pSS: detachment of the basement membrane, alterations of the apical microvilli and disorganization of the tight junctions separating the apical and basolateral poles [198]. Several studies have shown that SS labial SG (LSG) display significant increase in proteolytic activity of matrix metalloproteinases (MMPs) and higher expression of MMP-3 and MMP-9 exclusively in acinar and ductal cells [199]. Some of the cytokines synthesized by the inflammatory cells, acinar and ductal cells of SS LSG can induce increased MMPs expression [108,200]. In turn, high MMPs expression triggers a high level of remodelling activity in the basal lamina that enhances the vulnerability of SGEC to direct contact with cytotoxic inflammatory cells [201]. The disorganisation of the basal lamina of acini and ducts of LSG from patients with SS is the most frequent modification observed that positively correlates with the number of inflammatory cells within the gland.

## 4. Clinical Manifestations

Although often reduced to its sicca syndrome due to its tropism for glandular tissue, pSS remains a systemic disease that can affect virtually all organs. These clinical manifestations can be due to various mechanisms: dryness secondary to exocrinopathy, autoimmune epithelitis with periepithelial lymphocytic infiltration of target organs, associated organ-specific autoimmunity with specific autoantibodies, systemic manifestations linked to the presence of immune complexes or cryoglobulinemia and clonal lymphocytic expansion. Three-quarters of pSS patients will have at least one extraglandular manifestation, ranging from mild inflammatory arthralgia to life-threatening manifestations. The clinical manifestations can occur at diagnosis or during follow-up, even after more than 10 years, which must justify careful monitoring of patients. In general, the manifestations due to lymphocytic infiltration around an epithelium of a target organ have a stable and indolent course (e.g., sicca syndrome, renal tubular acidosis, pulmonary involvement) while the autoimmune disorders linked to immune complexes or autoantibodies have a more unpredictable course, with flares and remissions.

### 4.1. General Manifestations

More than half of pSS patients report disabling fatigue and non-restful sleep [202], partly related to poor sleep quality due to dryness, night pain and an increased prevalence of obstructive sleep apnoea [203]. Low-grade fever is found in 6% to 41% of pSS patients [204], while periodic fever is found more anecdotally [204]. Weight loss and night sweats may also be due to the systemic activity of the disease, autonomic involvement or lymphoma development. B symptoms—the triad of fever, night sweating and weight loss classically described in lymphomas—are found only in 15% of low-grade lymphomas associated with pSS [205].

### 4.2. Ocular Manifestations

Dry eye is a classic manifestation of pSS, part of the sicca syndrome affecting more than 95% of pSS patients. Patients can report inability to tear, foreign-body sensation, conjunctival inflammation, eye fatigue and decreased visual acuity. Ocular dryness can be complicated by keratoconjunctivitis sicca, blepharitis, bacterial keratitis or corneal ulcer [206]. Uveitis, episcleritis and orbital pseudotumor are rare but possible systemic manifestations [207].

### 4.3. Stomatologic Manifestations

Lymphocytic infiltration of SG generates exocrinopathy with hyposialia responsible for soreness, adherence of food to the mucosa, dysphagia, difficulties in speaking or eating, dental caries, tooth loss, periodontal involvement, lip dryness and nonspecific ulcerations and aphthae [206,208]. Oral candidiasis and angular cheilitis are mycotic complications related to the loss of antimicrobial action of saliva [209]. Parenchymal involvement can be complicated by recurrent parotid enlargement of infectious, lithiasic, inflammatory or lymphomatous origin [210]. SG may be the site of bilateral multicystic parotid masses and lymphoma.

### 4.4. Musculoskeletal Manifestations

Joint inflammatory manifestations are, after sicca syndrome, the most frequent manifestations of pSS (50% of patients) [211]. Patients may have arthralgia with inflammatory characteristics (morning stiffness > 30 min) or less frequently true symmetric polysynovitis mimicking rheumatoid arthritis (RA). Joint involvement of the pSS is generally moderate (<5 affected joints) and preferentially affects the small joints of the hands and upper limbs [211,212]. Joint involvement is conventionally non-erosive—except in case of an overlap with RA—but can be deforming (Jaccoud arthropathy) [211]. More rarely, pSS can be responsible for myositis. Finally, widespread pain is frequent—nearly 50% of pSS patients—resembling primary fibromyalgia [213,214].

### 4.5. Neurological Manifestations

Neurological manifestations of pSS are relatively frequent (18–45% of patients) and affect both the central and peripheral (sensitivomotor and autonomic included) nervous systems, with a higher prevalence of peripheral manifestations [215].

The peripheral manifestations are polymorphic and can be differentiated according to electromyographic examinations in mixed polyneuropathy, axon sensory polyneuropathy, sensory ataxic neuronopathy, axon sensorimotor polyneuropathy, pure sensory neuronopathy, mononeuritis multiplex or rarely chronic demyelinating polyradiculoneuropathy. The mechanisms mentioned are mainly lymphocytic infiltration of the dorsal root ganglia (for sensory ganglioneuronopathy), vasculitic lesions of the vasa nervorum and/or the presence of axon-specific autoantibodies. The cranial nerves can also be involved, essentially the trigeminal nerve by involvement of the Gasser ganglion (associated or not with a more extensive ganglionopathy) and the facial nerve (uni- or bilateral paralysis). The other cranial nerves are affected anecdotally. Finally, damage to non-myelinated fibres can be responsible of autonomic neuropathy or small-fibre neuropathy.

In the central nervous system, pSS may be responsible for encephalic or spinal manifestations, with stroke-like or Multiple Sclerosis-like damage secondary to cerebral vasculitis. Some demyelinating manifestations combining myelitis and optic neuritis are part of an associated neuromyelitis optica spectrum disorder (NMOSD), a condition linked to the presence of anti-aquaporin 4 autoantibodies. Neuro-pSS can also manifest as a recurrent aseptic lymphocytic meningitis. Rarely, the association of upper and lower motor neuron diseases resulting in an amyotrophic lateral sclerosis-like syndrome has been described during pSS.

Finally, cognitive dysfunction (“brain frog”), restless leg syndrome and psychiatric abnormalities are classically linked to pSS, but it is not clear whether these manifestations are reactive or directly linked to the pathophysiology of the disease.

### 4.6. Pulmonary Manifestations

The prevalence of clinically significant lung disease in pSS is 9–20% although subclinical manifestations can be found in more than 50% of patients by CT-scan or bronchoalveolar lavage findings. pSS exocrinopathy also affects the lower airways causing coughing, tracheobronchitis sicca, bronchial hyperresponsiveness (mimicking late-onset asthma), cylindrical bronchiectasis and bronchiolitis (mainly follicular bronchiolitis). This involvement of the small airway epithelium is rarely responsible for an obstructive ventilatory syndrome (11–14%) but can be complicated by recurrent pulmonary infections or atelectasis [216,217].

Nonspecific interstitial pneumonia (NSIP) and usual interstitial pneumonia (UIP) are the most frequent interstitial lung diseases (ILD) patterns during pSS, corresponding to 45% and 16% of cases respectively. Lymphocytic interstitial pneumonitis (LIP) arrives in 3rd position (15% of ILD cases) and can be considered as a more specific benign diffuse lymphoproliferative disorder of pSS, probably starting from the follicular bronchiolitis. It must be differentiated from pulmonary lymphoma, which is found in 2% of pSS-ILD. Other patterns such as organizing pneumonitis are less frequent (11%) or even rare such as pulmonary amyloidosis, alveolar haemorrhage, Langerhans’ histiocytosis, cavitary lung disease and/or combined pulmonary fibrosis and emphysema syndrome. However, presence of multifocal cysts on CT-scan should raise clinical suspicion for pSS-ILD [211,216,217].

Pleural involvement is rare. In fact, pSS manifests by pleurisy only in less than one percent of cases [207]. Shrinking lung syndrome occurs in extremely rare cases in pSS patients [218,219,220,221,222,223].

### 4.7. Dermatological Manifestations

Cutaneous involvement in pSS is relatively common and multiple manifestations are described such as xeroderma, eyelid dermatitis, annular erythema/subacute cutaneous lupus-like lesions and vascular purpura (caused by cutaneous vasculitis, urticarial vasculitis, cryoglobulinemia or hypergammaglobulinemic purpura of Waldenström) [211]. More rarely pSS can be responsible for cutaneous ulcer, livedo, erythema nodosum, panniculitis, amyloidosis or granuloma annulare [209].

### 4.8. Cardiovascular Manifestations

Raynaud phenomenon is the most frequent vascular manifestation, affecting 15% of patients [207]. Fortunately, cardiac manifestations such as pericarditis, pulmonary hypertension and cardiomyopathy are very rare, affecting <1% of pSS patients, respectively [207]. Cardiac rhythm disturbances have been described, secondary to ionic disorders, dysautonomia or direct impairment of the electrical conduction system of the heart [224,225].

### 4.9. Oeso-Gastrointestinal Manifestations

Dysphagia is a frequent complaint in pSS patients generally related to inadequate lubrication of the upper aerodigestive tract and food bolus resulting from hyposalivation. Oesophageal dysmobility is also mentioned in certain cases, explaining the lack of correlation between xerostomia and dysphagia [226,227]. Dyspepsia is frequent, occurring in 23% of pSS patients, and often linked to chronic atrophic gastritis where inflammatory infiltrates similar to those of the SG are found following tissue histological examination. Antibodies against parietal cells or intrinsic factor can be found, but pernicious anaemia remains rare [226]. Manifestations such as diffuse abdominal pain, diarrhoea or malabsorption can occur as part of a protein losing enteropathy or in case of overlap with Celiac disease [226,227]. Interestingly, pSS patients with Primary Biliary Cirrhosis overlap (PBC) are at higher risk of developing duodenal ulcers (85% of cases) [226]. The digestive tract can be the site of acute and serious complications in the context of cryoglobulinaemic vasculitis.

### 4.10. Pancreatic and Hepatobiliary Manifestations

The pancreas being an exocrine gland, it is not surprising to find cases of acute pancreatitis, chronic pancreatitis or pancreatic insufficiency in 0–7% of pSS patients. Moreover, 25% to 33% prevalence of chronic pancreatitis-like morphologic changes suggest that there are many asymptomatic cases [226]. Hepatomegaly is found in 10–20% of patients. Liver tests are disrupted in 10–50% of patients, usually mildly and with no particular clinical significance. pSS can be associated with Primary Biliary Cirrhosis (PBC)—another autoimmune epithelitis—or with autoimmune hepatitis (AH). Pseudolymphoma has been described to occur in liver like it may occur in salivary or lacrimal glands [226,227].

### 4.11. Uronephrologic Manifestations

Schematically, renal involvement linked to pSS can be divided into 3 groups: (1) tubulointerstitial nephritis linked to autoimmune epithelitis characterized by peritubular lymphocyte infiltration, (2) glomerulonephritis associated with immune complexes and (3) disorders linked to the presence of specific autoantibodies. According to different cohorts, about 5% of pSS patients have a renal involvement. However, this figure seems clearly underestimated if occult tubular involvement is systematically assessed [211,228].

Tubular involvement can be associated with dysfunction of any part of the renal tubule and can be responsible for polyuropolydypsic syndrome, low molecular weight proteinuria, aminoaciduria, euglycemic glycosuria, acidosis with normal anion gap, hypokalaemia that may be complicated by paralysis or disturbed heart rhythm, hypophosphoremia linked to increased phosphate excretion that may be complicated by osteomalacia, nephrocalcinosis or the formation of recurrent kidney stones [228,229]. More anecdotally, acquired Gitelman or Bartter syndrome has been described, possibly linked to the presence of specific autoantibodies targeting transporters (ie NaCl co-transporter in Gitelman syndrome) [228,230]. Glomerular disease occurs later in the history of the disease and most often corresponds to a mesangioproliferative glomerulonephritis (MPGN) caused by the deposition of immune complexes, usually cryoglobulinemia, which should be looked for [211,228].

Interstitial cystitis is a chronic inflammatory disease of the bladder that can be found in pSS patients. This rare manifestation is characterized by complaints such as pollakiuria, lower abdominal pain, urinary urgency, painful micturition, haematuria and dysuria [231]. Interstitial cystitis can be complicated by bilateral hydronephrosis and obstructive renal failure [231].

### 4.12. Haematological Manifestations

Anaemia is present in 20% of pSS cases, usually normochromic normocytic, of various mechanisms: anaemia of chronic disease or haemolytic, more rarely secondary to aplastic or pernicious anaemia or myelodysplastic syndrome [232,233]. Leukopenia is found in 15% of patients and most often corresponds to lymphocytopenia. Agranulocytosis is rare. Thrombocytopenia is found in 15% of patients, of peripheral origin, whether or not involved in Evans syndrome [232,233]. Rare cases of Thrombotic Thrombocytopenic Purpura (TTP) [234,235,236] and Hemophagocytic lymphohistiocytosis (HLH) [237] have been described.

Reactive multiple lymphadenopathy is possible, statistically associated with the presence of synovitis [212]. The intense stimulation of B cells explains the occurrence of hypergammaglobulinemia, hyperviscosity syndrome, monoclonal gammapathy, cryoglobulinemia and amyloidosis [232,238]. The formation of immune complexes leads to complement fraction consumption.

CD4-Lymphocytopenia is mainly found in anti-Ro-SSA positive patients and is associated with an increased risk of non-Hodgkin’s lymphoma (NHL) [232]. NHL has a prevalence of 4.3% in pSS patients [205]. Schematically, pSS-associated NHL can be divided into two main categories: the first has an indolent course and is dominated by the extranodal marginal zone (MZ) B cell lymphomas of MALT-type, and the second corresponds to the high-grade lymphomas such as de novo or secondary diffuse large B cell lymphoma (DLBCL). In pSS patients, MALT lymphomas are indolent diseases characterized by a good performance status, small tumour burden and infrequent B symptoms. They are preferably located in one or more extranodal sites such as SG, stomach, nasopharynx, lung, liver, kidney, orbit and skin [205]. It is interesting to note that almost all of these sites are organs involved in autoimmune epithelitis. Locoregional nodal involvement can be observed while bone marrow infiltration is rare. DLBCL are aggressive and have a poor prognosis. A certain proportion of them probably come from a transformation from a low-grade lymphoma. NHL mainly occurs in pSS patients with cryoglobulinemia, palpable purpura and C4 fraction consumption [205].

### 4.13. Ear–Nose–Throat (ENT) Manifestations

ENT complaints are common (40–50%) in pSS patients but objective fibroscopic abnormalities are less frequent (20%) [239]. Exocrinopathy can generate rhinitis sicca—reported by about 40% of pSS patients—which is a source of discomfort, nasal crusting, sinusitis, epistaxis or smell and taste disorders [240]. pSS patients are more likely to develop laryngopharyngeal reflux (LPR) because oesophageal involvement impairs anti-reflux mechanisms. LPR—in addition to pharyngitis sicca—manifests itself through various ENT complaints such as dysphonia, throat pain, chronic throat clearing or Eustachian tube dysfunction [241].

As with other systemic vasculitides, pSS may be responsible for sensorineural hearing loss or chondritis [242], responding to corticosteroid treatments. In an appealing way, pSS is associated with a sensorineural hearing loss in a significant proportion of patients, mainly affecting high frequencies, but whose clinical impact is not obvious [243].

### 4.14. Gynaecological and Obstetrical Manifestations

pSS does not have a negative impact on fertility, but chronic pain and vaginal dryness can be the cause of dyspareunia having a negative impact on the sexuality of female patients [244]. During pregnancy, pSS can be responsible for two rare but classic manifestations: autoimmune congenital heart block and neonatal lupus [245,246,247]. These two manifestations are linked to the transplacental passage of anti-Ro/SSA autoantibodies. Congenital heart block occurs in 2% of anti-Ro/SSA positive pregnancies but with a 10 to 20% risk of recurrence in subsequent pregnancies. More rarely, neonatal lupus can be associated with endocardial fibroelastosis, valvular malformations or septal defects. Neonatal lupus—affecting one fifth of anti-Ro/SSA positive pregnancies—is characterized by an erythematous rash and photosensitivity that can be associated with hepatic, haematological and neurological involvement. Compared with healthy pregnancy, patients with pSS had significantly higher chance of pregnancy loss or neonatal death. However, there were no significant associations between pSS and premature birth, spontaneous or artificial abortion or stillbirth [248]. These data should be taken with caution because they are based on a limited number of heterogeneous—and not necessarily recent—studies.

## 5. Diagnosis Workup

### 5.1. Diagnosis Versus Classification Criteria

Faced with one or more compatible manifestations, the diagnosis of pSS must be evoked and investigated. Making a diagnosis is the basis of medical care. For the patient, it represents the end of questioning and diagnostic wandering. For the physician, the diagnosis makes it possible to clarify the management. Finally, for the researcher, the diagnosis makes it possible to create homogeneous groups around a consensus definition. Unfortunately, there is no single diagnostic test to confirm the diagnosis of pSS. Due to its protean and willingly insidious presentation, pSS is sometimes difficult to recognize and may delay diagnosis by more than 10 years. Sicca syndrome, fatigue and unspecific musculoskeletal pain can be wrongly taken for manifestations of age, anxio-depression or perimenopause in people with pSS. Systemic manifestations can sometimes precede sicca syndrome, resulting in an “occult pSS” [249]. For these various reasons, the gold standard for individual diagnosis of pSS remains the opinion of an expert clinician. To allow the study of the disease in groups of pSS patients, several consensuses have defined classification criteria allowing a common definition of what pSS is. The 3 most recent sets of classification criteria are presented in Table 2. By definition, classification criteria are specific but may lack sensitivity and should not be used blindly as diagnostic criteria but as a guide in clinical practice.

### 5.2. Sicca Syndrome and Glandular Assessment

The investigation for objective dysfunction of the salivary and lacrimal glands is useful for the diagnosis and symptomatic management of the patient. Anatomical or functional imaging can be used to assess changes in the major SG during pSS.

The evaluation of dry eyes requires a simple ophthalmological examination. The Schirmer test consists of positioning a small strip of filter paper inside the inferior fornix of each eye. The eyes are then closed for 5 min. After this time, the strips are removed, and the amount of tears absorbed by capillarity is measured in millimetres from the edge of the strip in contact with the ocular surface. Dryness is significant if ≤5 mm/5 min. The evaluation then continues with the evaluation of the stability of the tear film by the Break-up Time (BUT) and the search for conjunctival or corneal lesions linked to dryness (keratoconjunctivitis sicca). These various tests use the slit lamp and the ocular instillation of dyes. BUT is measured by placing a drop of fluorescein in each eye and measuring the time during which the coloured tear film uniformly covers the ocular surface, before the appearance of dry spots. A tear BUT test of less than 10 s (averaged over 3 testings’) is considered pathological but is not specific of pSS manifestations. Finally, damage to the conjunctiva and cornea is highlighted by ocular surface staining techniques (fluorescein and lissamine green) [253]. The anomalies are scored using standardized scores: van Bijsterveld scale or the SICCA Ocular Staining Score (OSS). Respective cut-offs of ≥4 and ≥5 correspond to pathological situations suggestive of pSS. Those tests are more specific of pSS than Schirmer and Break-up time tests. Rose Bengal dye is no longer used because of its poor tolerance and local toxicity.

The evaluation of hyposalivation can be easily performed by sialometry. In its simplest form, sialometry consists of measuring the Unstimulated Whole Salivary Flow rate (UWSF) and the Stimulated Whole Salivary Flow rate (SWSF). UWSF is performed by asking the patient—fasted for minimum 2 h—to passively drain all the saliva produced in a tared jar for 15 min. The jar is then weighed and the saliva volume estimated. UWSF less than 0.1 mL/min is considered pathological (normal range 0.3–0.4 mL/min). UWSF represents a minor classification criterion. SWSF is measured in the presence of mechanical stimulation. SWSF can be measured using the Saxon test or Gum test protocols. Saxon test is performed by asking the patient to chew for 2 min a tared compress which will then be weighed. Gum test is performed as USWF, but in this case, the patient chews chewing gum and then spits saliva in a container. A diagnosis of hyposalivation is made if SWSF is ≤0.5–0.7 mL/min (normal range 1.5–2.0 mL/min). It is also possible to measure the salivary flow specific to each major SG by aspiration or cannulation. However, these techniques are of little use to the rheumatologist and especially uncomfortable for the patient.

Radiosialography is an X-ray imaging technique requiring the retrograde injection of a contrast solution into the excretory ducts of the major SG. This technique indirectly highlights glandular damage by studying changes in the “tree structure” of the excretory ducts [254]. Given the invasive nature and the complications of this technique, it has been abandoned in favour of other non-invasive techniques.

SG scintigraphy (SGS) studies the uptake, the concentration and the basal or stimulated secretion of a radioactive tracer by the parotid and submandibular glands following an infusion of Technethium-99 pertechnetate. SGS interpretation is mainly based on Schall’s classification [255], a qualitative score classifying anomalies in 4 grades—from grade 1 (normal) to grade 4 (the total absence of uptake and mouth activity). With ≥3 as cut-off, sensitivity and specificity are 54–87% and 78–98%, respectively [256]. Salivary scintigraphy is one of the classification criteria of 2002 for pSS but has disappeared from the most recent classification criteria of 2016. An abnormal scintigraphy makes it possible to objectify a dysfunction of the SG but does not allow etiological diagnosis as no image is specific of pSS. However, it may be of interest for treatment: if the examination shows SG with normal uptake but with a major dysfunction of excretion (possibly due to an autonomic disorder), the patient could benefit from a sialagogue treatment. In case of a scintigraphy demonstrating no uptake of the tracer, the parenchyma is probably totally destroyed and a sialagogue treatment will be useless.

Ultrasound is a simple, non-invasive way to assess the parenchyma of parotid and submandibular glands for diagnostic and prognostic evidence for pSS. Mode-B ultrasound using a high frequency linear probe allows characterization of size, homogeneity, presence of hypo-/anechoic areas, hyperechoic bands and clearness of SG borders. These different items were included in several diagnostic scores [257]. The OMERACT group, in an attempt to standardize, developed in 2019 a semi-quantitative scoring (0–3) based on the presence of hypoechoic/anechoic zones within the parenchyma of the parotid and submandibular glands [258]. A score ≥ 2 is abnormal and suggestive of pSS. At present, SG ultrasound (SGUS) is not part of classification criteria but may well be in the future [259]. Unfortunately, correlations between histological abnormalities (lymphocytic infiltration, diseased parenchyma or ductal ectasia/cysts) and SGUS lesions have not been corroborated [254]. SGUS scores improvement after treatment with Rituximab prove that part of the abnormalities are correlated with the disease activity and not only damage accrual [260,261]. To date, there is currently insufficient evidence to use SGUS as a prognostic or treatment response factor. Thanks to its high spatial and contrast resolution, low cost and accessibility, SGUS has replaced MRI in the diagnosis of the pSS patient.

### 5.3. Labial Minor SG Biopsy

The minor SG biopsy (MSGB) is a simple procedure that can be performed with little equipment. Several biopsy techniques have been described in the literature [262,263]. After disinfection, the reappearance of small drops of saliva makes it possible to identify the accessory SG at the level of the lateral third of the lower lip. The mucosa above these glands is anesthetized with an injection of lidocaine. The mucosa is then opened with a scalpel over 5–10 mm and the glands removed with forceps. The individualization and extraction of the glands is made easier by the hydrodissection that occurs during local anaesthesia and by the eversion of the lip. Lobules are herniated towards the surface of the wound by the application of pressure—digital or instrumental—on the external part of the lip. For quality concerns, the removal of 4–6 glands—allowing the study of minimum 8 mm^2^ of glands—is recommended [264]. A parotid biopsy is only exceptionally performed because technically more complex with a theoretical risk of damage to the facial nerve, for a diagnostic contribution identical to MSGB based on focus-score. On the other hand, the detection of lymphoepithelial lesions and early stage lymphomas—having a prognostic value—is more frequent/easier to detect on parotid biopsies [263].

The central element of MSGB pathology is the presence of clusters of more than 50 mononuclear cells (mainly lymphocytes) called foci. These foci in periductal or perivascular areas adjacent to normal acini are counted, reported to the area investigated and expressed as a Chisholm–Mason score [265] or a Focus-score [266]. Compared to the initial descriptions of those scores, some experts recommend counting all foci, including those associated with areas of fibrosis or atrophy, for fear of changing the Focus-score [264]. The Focus-score corresponds to the average number of foci per 4 mm^2^ of gland. It goes from 0 to 12, 12 corresponding by convention to the coalescence of the foci. The Chisholm score ranks chronic sialadenitis from 0 to 4. Grade 0 corresponds in the absence of infiltration; grade 1 corresponds to a slight infiltration of mononuclear cells, however not forming a focus; grade 2 corresponds to the presence of an infiltrate of mononuclear cells organizing in foci but whose density is <1 focus per 4 mm^2^; grades 3 and 4 correspond to the presence of 1 or > 1 focus per 4 mm^2^, respectively. The presence of focal sialadenitis characterized by a Focus-score ≥ 1 (Chisholm grade ≥ 3) is a major diagnostic argument for pSS and is included in the different classification criteria. Due to its sensitivity and specificity >80% and its significant positive predictive value [267], the presence of a chronic focal sialadenitis (Focus-score ≥ 1) is particularly useful in the diagnosis of early pSS, even with specific manifestations and autoantibodies negativity [249].

Although not part of the classification criteria, other anomalies can be described: fibrosis, acinar atrophy, ectasia or metaplasia of the excretory ducts, histiocytic granulomas, presence of germinal centre-like structures, lymphoepithelial or myoepithelial sialadenitis (LESA/MESA) [268,269]. LESA/MESA are characterized by lymphocytic infiltration of ducts and basal cell hyperplasia, resulting in a multilayered epithelium. In addition, pathology allows differential diagnosis with sarcoidosis, IgG4-related disease, amyloidosis and lymphoma. Finally, MSGB provides information on the patient’s prognosis: a Focus-score ≥3 and the presence of germinal centre-like structures or LESA/MESA are associated with more severe disease and an increased frequency of local and systemic manifestations, including lymphoma. For this reason, we recommend doing MSGB even if the diagnosis can be made based on anti-Ro/SSA positivity with objective sicca syndrome.

The parotid biopsy has fallen somewhat into disuse due to the ease of performing a minor SG biopsy with equivalent diagnostic performance. On the other hand, the possible discrepancies with MSGB [270,271], the possibility of early detection of lesions associated with a poor prognosis, the possibility of biopsying the same gland again to monitor the disease and the possibility of correlating it with SGUS semiology make parotid biopsy a tool that would need to be reassessed in the future [263].

### 5.4. Antinuclear Antibodies (ANA) Profile

The other major element in the diagnosis of pSS is the presence of anti-Ro/SSA and/or anti-La/SSB autoantibodies. The Ro/La system is a heterogeneous antigenic complex, composed by three different proteins (52kDa Ro, 60kDa Ro and La) and four small RNAs particles [272]. The search for antinuclear antibodies (ANA) by Immunofluorescence (IF) on HEp-2/HeLa cells is therefore an important element in the diagnosis of pSS. ANA is positive in 70% of pSS patients, usually with a fine speckled fluorescence [273]. Anti-Ro/SSA and/or anti-La/SSB autoantibodies are identified in 50–90% and 25–60% of patients, respectively [274]. It should be borne in mind that the Hep-2 cells do not sufficiently express Ro/SSA antigen, explaining the fact that 10% of patients anti-Ro/SSA-positive in ELISA have negative ANA in IF on HEp-2 cells [274]. Therefore, in case of suspicion of pSS, it is necessary to request the anti-Ro/SSA antibodies identification by ELISA, even in the presence of a negative ANA IF screening. Two types of anti-Ro/SSA autoantibodies can be differentiated: anti-Ro52 and anti-Ro60 [272]. Anti-Ro52/SSA have no specific ANA fluorescence staining pattern (might even exhibit a cytoplasmic pattern [274]), is precipitin negative and is not detected by ELISAs based on natural SSA/Ro. Ro52+ Ro60+ patients are likely to have pSS while Ro52+ Ro60- patients are not [275]. Isolated anti-Ro52/SSA positivity is statistically linked to primary myositis and systemic sclerosis. On the other hand, anti-Ro52/SSA and anti-La/SSB have the highest relative risks of congenital heart block in offspring from anti-Ro/SSA positive patients because these two antigens are expressed in foetal cardiac tissue from the 18th to 24th week [272]. Anti-La/SSB is mainly found in the presence of an anti-Ro/SSA, evoking a mechanism of epitope spreading. In only 2–3% of cases, pSS patients present with an isolated Anti-La/SSB antibody [276,277]. The presence of another ANA pattern or the identification of “atypical” ANAs can allow the identification of a secondary SS, an overlap with another systemic disease or a specific pSS subgroup [159]. The prognostic implication of these antibodies is discussed in the prognosis section.

### 5.5. Blood Workup

In addition to ANA testing, the initial blood workup for suspected autoimmune systemic disease includes a complete blood count; a coagulation profile with antiphospholipid panel; urea/creatinine dosage and urine sediment and 24-h urine protein or urine protein/creatinine levels; Na^+^/K^+^/HCO_3_^−^/Cl^−^/Uric Acid levels to investigate renal tubulopathy; hepatic enzymes levels; creatine phosphokinase (CPK) to investigate myositis; C3/C4/CH50 levels, Rheumatoid Factor (RF), Cyclic Citrullinated Peptide (CCP) antibodies, Coombs test; serum protein electrophoresis and total IgG, IgM and IgA levels to investigate presence of polyclonal hypergammaglobulinemia and/or monoclonal gammapathy; HCV serology; VDRL/TPHA; free T4 levels, TSH, anti-thyroid peroxidase, anti-thyroglobulin, anti-mitochondrial, anti-smooth muscle, anti-gastric parietal cell antibodies in case of associated auto-immune diseases. Hypergammaglobulinemia and lymphopenia are classically described during pSS. Their presence may be an additional argument, but their diagnostic performance is not known.

### 5.6. Sjögren’s Syndrome Differential Diagnosis

Classically all disorders manifested clinically by sicca symptoms, glandular enlargement and/or rheumatic/systemic manifestations fall under the differential diagnosis of pSS (Table 3). However, a rational and pragmatic approach often leads to the correct diagnosis [278].

### 5.7. Primary versus Secondary Sjögren’s Syndrome

It is classic in medical nosology to describe the isolated and idiopathic form of a disorder as “primary” and to qualify as “secondary” the forms associated with specific causes or entities. SS is no exception. Historically, this dichotomy differentiated pSS patients from patients suffering from RA complicated by sicca syndrome. Subsequently, “secondary SS” (sSS) extended to other connective tissue diseases (e.g., SLE and Systemic Sclerosis (SScl)) and autoimmune diseases (e.g., primary biliary cirrhosis, thyroiditis and vasculitis) [279]. This nomenclature has also been indirectly “ratified” in AECG Classification Criteria from 2002 [250], classifying as “sSS” patients with another well-defined major connective tissue disease and at least one dry symptom (ocular or buccal) and 2 out of 3 signs of exocrine dysfunction (MSGB, SG signs or ocular signs in Table 2).

In light of current data, this dichotomy seems obsolete and should be reviewed. While polyautoimmunity and overlap syndromes are currently recognized, one can wonder why SS is still considered a second-class disorder.

Based on the examination of salivary gland biopsies of 34 RA patients with sicca symptoms, two phenotypes can be differentiated [280]. One group of patients presented a phenotype characterized by mild salivary gland lesions and negative autoantibody. Histologically, minor SG biopsies display increased prevalence of antigen-presenting cells and CD8+ T cells, decreased presence of B cells, and “non-activated” epithelial cells (based on the expression of HLA-DR and co-stimulation proteins D80/B7.1). A second group of patients presented a phenotype characterized by glandular manifestations and/or auto-antibodies positivity. Their minor SG biopsies demonstrated CD80/B7.1 overexpression and low frequency of S100+ cells, correlated with the positivity of anti-Ro/SSA autoantibodies and/or focus score ≥ 1. Both groups had an historical RA-sSS and an RA-pSS overlap, respectively. In this study, compared to RA patients without sicca symptoms, RA-sicca patients statistically present more Raynaud’s phenomenon, SG enlargement, palpable purpura and renal, lung and liver involvement. They displayed more frequent ANA, anti-Ro/SSA autoantibodies and RF positivity. The published data do not allow us to know if these manifestations are over-represented in the second group.

From a serohistological point of view, there is no difference in terms of anti-Ro/SSA positivity, anti-La/SSB positivity and SG infiltration between a pSS alone and an sSS associated with a SLE [281] or SScl [282]. It therefore seems more like an overlap than a so-called sSS. On the other hand, as for RA patients, SS overlap modifies the associated clinical phenotype. Compared with SLE-alone patients, patients with SLE-SS overlap are older and had a higher frequency of Raynaud’s phenomenon, anti-Ro/SSA positivity, anti-La/SSB positivity and rheumatoid factor. They also had a significantly lower frequency of renal involvement, lymphadenopathy and thrombocytopenia [281]. Compared with SScl-alone patients, patients with SScl-SS overlap seem less at risk of serious complications from SScl namely lung fibrosis, pulmonary artery hypertension and scleroderma renal crisis [282].

To summarize, “secondary SS” is to be banned from our vocabulary [283] or—at a pinch—redefined very restrictively for some exocrine involvement occurring in rheumatoid arthritis not corresponding to a real SS, if such an entity exists. Moreover, “secondary SS” has disappeared from the classification criteria of 2012 and 2016. The patient has or does not have (p)SS, which may or not be associated with other autoimmune diseases, reflecting common etiopathogenic pathways. In this way, the clinician avoids three pitfalls: (1) minimizing the SS-related symptoms, which decrease the quality of life of the patients; (2) forgetting that overlap may change the clinical phenotype and (3) forgetting the risk of lymphoma. Unfortunately, pSS overlap syndromes had been under-recognized, under-researched and possibly under-treated in the past because of the historical label of “secondary SS” and their exclusions from the majority of clinical trials [284]. Their management is therefore based on the clinician’s expertise, patient choices, best evidence and practice for the management of all associated diseases. To better individualize pSS in the future, it would be necessary to be able to move from a clinical definition to a molecular or even epigenetic signature.

## 6. Prognosis

Once the pSS diagnosis is made, treatment and medical decisions will be based on the expected course of the disease and its impact on the patient’s life. This burden can be summarized in “5D”: Death (mortality), Disease activity, Damage accrual, Discomfort (pain and sicca symptoms) and Disability. To assess the effect of therapeutic interventions on the natural history and functional repercussions of the disease, scores that can be used as clinical outcomes in trials have been developed.

### 6.1. Death

Although overall pSS mortality is low and similar to the general population [285], a subgroup of patients will have a poorer vital prognosis. The excess mortality observed in such subgroup of patients is generally attributed to the development of lymphoma or to uncommon but severe visceral involvement. The leading causes of mortality in pSS patients are cardiovascular events, followed by solid-organ and lymphoid malignancies and infections [285]. Risk factors associated with increased mortality are advanced age at diagnosis, male sex, parotid enlargement, abnormal parotid scintigraphy, extraglandular involvement, vasculitis, anti-SSB positivity, low C3 and C4 and cryoglobulinaemia [285].

pSS is associated with increased risks of overall cancer (pooled RR 1.17 to 1.88), non-Hodgkin lymphoma (NHL) (pooled RR 8.53 to 18.99) and thyroid cancer (pooled RR 1.14 to 4.03) [286,287]. Biomarkers associated with the development of lymphoma are mainly signs associated with exuberant B cell proliferation and immune-complex production [288,289,290]: parotid swelling, Focus-Score ≥3, germinal centre-like lesions, skin vasculitis or palpable purpura, complement consumption (Low C3, C4 or CH50), presence of cryoglobulinemia or monoclonal paraproteinemia, rheumatoid factor, increased β-2 microglobulin, lymphocytopenia, hypoglobulinemia, lymphadenopathy or splenomegaly and head and neck irradiation.

### 6.2. Disease Activity

Disease activity may be defined as the functional or structural changes in an organ related to inflammatory burden of the disease and are reversible under treatment. As in other inflammatory diseases, disease activity can fluctuate over time and progress between relapses and remissions. A significant proportion of pSS patients—nearly 50–70%—display a systemic manifestation at the time of glandular onset or within 6 months, mainly lymphadenopathy/splenomegaly, non-erosive arthritis and neurologic involvement [291]. The long-term study of the Antonius Nieuwegein Sjögren (ANS) cohort revealed that, within 10 years of diagnosis, 30.7% of the 140 patients included in this study developed an associated extraglandular or autoimmune manifestation such as polyneuropathy, interstitial lung disease, arthritis, discoid or subacute cutaneous lupus erythematosus (LE) and Hashimoto’s disease [292]. The presence of cryoglobulinemia is associated with an increased risk of developing a systemic manifestation [211,292]. On the other hand, presenting widespread pain seems to be a “protective phenotype” [292].

Currently the European League Against Rheumatism (EULAR) SS disease activity index (ESSDAI) score has been used to quantify the inflammatory systemic activity of the disease. Within ESSDAI, clinical or biological manifestations are classified as “low” (1 point), “moderate” (2 points) or “high activity” (3 points) in 12 domains. To calculate the ESSDAI score, the value of the highest level of activity for each domain is multiplied by the domain weight (1 to 6) and then added together. The maximum theoretical ESSDAI score is 123. Minimal clinically important improvement was defined as an improvement of at least three points. More recently, ClinESSDAI score, a variant of the ESSDAI score without the biological domain, has also been used [293] (Table 4).

However, it should be borne in mind that (clin) ESSDAI score does not investigate all of the possible events related to pSS. Out of 6331 patients included in the international register “The Big Data Sjögren Project Consortium” [207], 1641 patients (26%) had at least one non-ESSDAI systemic manifestation on a predefined list of 26 organ-specific features not currently included in the ESSDAI classification. Patients with non-ESSDAI manifestations are patients with higher systemic activity than patients without non-ESSDAI manifestations (mean ESSDAI 10.3 vs. 5.5, *p* < 0.001).

Patients with significant systemic activity are generally patients with early onset disease, antinuclear antibodies (ANA) positivity with a higher frequency of anti-Ro/SSA (with or without anti-La/SSB), low C3, low C4 and cryoglobulinemia [154,276,277,298]. Children of anti-Ro/SSA positive mothers are at risk of specific neonatal complications such as neonatal lupus and congenital heart block [277]. Paradoxically, patients with higher disease activity are less disabled by sicca syndrome or widespread pain [276,277]. Conversely, patients with late-onset seronegative disease will mainly present a more disabling sicca syndrome but fewer systemic manifestations linked to the activity of the disease [277]. Finally, isolated anti-La/SSB positivity occurs in only 3% of pSS patients and is associated with an intermediate phenotype between Ro/SSA positive- and seronegative patients [277]. Thus, systemic complications could appear many years after initial pSS diagnosis and justify long-term surveillance, especially in cryoglobulinemia or “high risk” phenotype patients.

The immunological profile of pSS highlights the presence of atypical ANA—12% of cases [299]—or other specific autoantibodies. A subset of pSS patients with anti-centromere positivity develops a clinical phenotype overlapping between SS and systemic sclerosis with a higher age, more frequent Raynaud’s phenomenon and keratoconjonctivitis sicca and a lower proportion of anti-Ro/SSA and anti-La/SSB, rheumatoid factor, leukocytopenia and hypergammaglobulinemia [159,299]. In most cases, a minority of these patients appear to progress to an authentic systemic sclerosis. Anti-Cyclic Citrullinated Peptides (anti-CCP) positivity—present in 3–10% of patients—is associated with a greater frequency of joint manifestations or with overlap with rheumatoid arthritis (RA) [159,277]. The presence of anti-mitochondrial antibodies (1.7–13%) and anti-smooth muscle/anti-liver kidney microsomal antibodies (30–62%) is associated with overlap with primary biliary cirrhosis and autoimmune hepatitis [159].

### 6.3. Damage Accrual

Disease damage may be defined as the addition over time of irreversible functional or structural changes resulting from disease activity, iatrogenic treatments or co-morbidities.

Two scores exist to quantify damage related to pSS: SS Disease Damage Index (SSDDI) [296] and SS Damage Index (SSDI) [297]. SSDDI is composed of a list of 18 irreversible damages affecting 6 organ-domains (oral, ocular, neurologic, pleuropulmonary, renal and lymphoproliferative), divided into 9 items weighted for severity. SSDI is an unweighted checklist of 27 items divided into 3 lists: ocular damage, oral damage and systemic damage. Systemic damage is further subclassified into 7 areas: neurological, renal, pulmonary, cardiovascular, gastrointestinal, musculoskeletal and malignancy (Table 4).

In a retrospective study using 148 pSS patients attending the UCLH Sjögren’s clinic followed for 10 years, Krylova et al. revealed that 28.3%, 36.7% and 45% of patients displayed SSDI damage (excluding oral damage that was not assessed in the study) after 1, 5 and 10 years of disease, respectively [300]. Items most involved are in the ocular domain, parotid swelling and malignancy. These results suggested that pSS patients accumulate less damage—calculated on different scores—over time than lupus patients, who have a greater inflammatory burden and use of immunosuppressive treatments [300].

Another retrospective study using 155 pSS patients showed that the total increase of patients with damage was 28% after 1 year, 44% after 3 years, 74% after 5 years and 83% at 10 years, with a good correlation between SSDDI and SSDI [301]. More specifically, teeth loss and/or caries, salivary flow impairment, corneal ulcers and tear flow impairment were reported in 49.5%, 34%, 22.6% and 11% of patients, respectively. Unsurprisingly, systemic damage—observed in 13.5% of patients—was correlated with basal ESSDAI, low C4 and lymphopenia. In the same way, persistent SG swelling—detected in 14% of patients—was associated with (bio)markers of systemic activity and B cell proliferation (lower age at diagnosis, anti-Ro/SSA positivity, cryoglobulinemia, low C4, hypergammaglobulinemia and lymphopenia). Lymphoproliferative disorders were detected in 4.5% and malignancy in 9% of cases at 10 years post-diagnosis [301].

### 6.4. Discomfort and Disability

SS can be disabling and associated with significant functional status impairment related to oral and/or ocular dryness, systemic activity, pain, fatigue and daytime somnolence, anxiety and depression symptoms [302,303,304]. Objective assessments of sicca syndrome correlated poorly with symptoms and remain generally stable over time [305]. Besides the associated symptoms, sicca syndrome also has a negative impact on smell, taste, pruritus, voice, swallowing and sexual function [306,307]. Fatigue and pain are both correlated with reduced quality of life and psychological distress [307]. Patients with widespread pain—34.9% of the cohort—were more frequently negative for anti-La/SSB, more frequently seronegative for all autoantibodies (ANA/SSA/SSB/RF) and had statistically fewer extraglandular manifestations in a Dutch study including 83 patients [308]. Another Italian study on 100 pSS patients demonstrated a prevalence of widespread pain of 22%, a phenotype statistically associated with fewer systemic and immunological manifestations (hypergammaglobulinemia, rheumatoid factor, focus-score ≥ 1) [309]. A subset of pSS patients therefore seem to develop a clinical phenotype with lower visceral involvement but with significant morbidity linked to glandular manifestations and a significant psychosomatic burden [302,310], bringing them closer to the notion of “Sicca Asthenia Polyalgia (SAP) Syndrome” [311,312,313]. At diagnosis, one in 4 patients is unable to work. This figure increases to more than 1 in 3 at 1 year. Work disability at 2 years is 40% and is related to fibromyalgia pattern, age and incapacity for work at diagnosis [314]. pSS has a high individual and societal cost, especially due to dental cost, symptomatic therapies and disease compensation [307].

EULAR SS Patient Reported Index (ESSPRI) is a consensus index calculated as the mean of 3 visual analogue scales (VAS)—self-assessment of dryness, (limb) pain and fatigue—allowing easy measurement of patients’ symptoms in pSS [295]. By convention, patient-acceptable symptom state was defined by an ESSPRI <5/10 and the minimal clinically important improvement by a decrease of at least one point or 15%. The ESSPRI score is correlated with the Patient Global Assessment [PGA] [295] and with more complex and time-consuming scores such as the Profile of Fatigue and Discomfort [PROFAD] [295], Sicca Symptoms Inventory [SSI] [295], Health Assessment Questionnaire [HAQ] [315], Short Form 36 health survey [SF-36] [302], time trade-off values [TTO] and EuroQol5D VAS [316,317]. Very interestingly, a study using baseline data from 120 patients included in the TEARS study revealed that—even if there is a small correlation between ESSPRI and ESSDAI—ESSPRI is the only determinant associated with the quality of life score SF-36 in a multivariate model [318]. The ESSPRI score is therefore a good clinical screening and monitoring tool as well as a good surrogate endpoint to study the effectiveness of therapeutic interventions on pSS associated “Sicca Asthenia Polyalgia” Syndrome (Table 4).

It is therefore important, a fortiori in mild cases with low activity score but disabling sicca syndrome, to focus on improving the quality of life of patients through attentive and multimodal symptomatic management and to offer a multidisciplinary management program for the most disabled.

## 7. Therapeutic

Despite a better understanding of its pathophysiology, treatment of SS remains disappointing and essentially palliative. Systemic activity is treated by immunosuppressant drugs, based on scarce evidence. Manifestations linked to damage caused by local or systemic activity of pSS should be identified because they are by definition irreversible and cannot therefore be improved by immunosuppressive treatments. In the last 5 years, pSS management has been addressed by guidelines from EULAR [210], British Society of Rheumatology and National Institute for Health and Care Excellence (NICE) [319], Brazilian Society of Rheumatology [320], Research Team for Autoimmune Diseases [321] and Sjögren’s Syndrome Foundation [322]. The main principles for care are summarized below.

### 7.1. Sicca Syndrome and Non-Visceral Manifestations

Despite the dysimmune origin of the disease, no immunosuppressive treatment has demonstrated sufficient efficacy associated with a satisfactory risk–benefit balance in the treatment of sicca syndrome and non-visceral aspecific manifestations (non-inflammatory widespread chronic pain, fatigue). Treatment is mainly focused on symptom management and prevention or treatment of complications resulting from exocrinopathy (Table 5).

Therapeutic approach to oral dryness must be driven by baseline objective and subjective severity of hyposialia and xerostomia. To this end, current guidelines recommend evaluating baseline SG function by measuring unstimulated (UWSF) and stimulated salivary flow (SWSF) or using salivary scintigraphy. Subjective xerostomia impact is captured by a simple Visual Analogue Scale, as part of the ESSPRI score. EULAR guidelines propose an algorithmic approach to the management of dry mouth: patients with an UWSF < 0.1 mL/min are categorized based on their SWSF as mild (>0.7 mL/min), moderate (0.1–0.7 mL/min) or severe dysfunction (<0.1 mL/min). Self-care advice and non-pharmacological stimulation are proposed to mild cases as first line therapy [210]. Pharmacological stimulation (pilocarpine per os or as a mouthwash, cevimeline per os) is the treatment of choice in moderate cases (with residual SG function) or in mild dysfunction patients who failed to respond to basic recommendations, in addition to first line therapy. Saliva substitutes are reserved for patients with no residual function or as a third line treatment in non-responding patients.

The stomatological complications of exocrinopathy affecting the SG are cavities formation, periodontal disease, candida infections and glandular swellings linked to abscess or to a lithiasic disease. It is therefore strongly recommended that patient adopts impeccable dental hygiene and be evaluated at least 2 times per year by a dental professional. Local fluoride-based treatments can be administrated. Candida simple infection (visible white plaques) are treated with Nystatin mouthwash for 7 days. One-week prophylactic treatment may be repeated every 8 weeks in the event of recurrence. Erythematous infection of tongue or oral cavity is treated with Fluconazole 50 mg for 10 days. Angular cheilitis is treated with Miconazole topically on each side of the mouth for 2 weeks. Presence of abscess or lithiasic involvement can be treated with antibiotic treatment and stomatologist involvement is indicated. If no infectious or mechanical cause is found in case of gland swelling, a distinction must be made between primary neoplasia, systemic activity of the disease (as scored in ESSDAI, treated by glucocorticoid in loco by sialendoscopy, per os or intra-muscular) and the appearance of a lymphomatous complication.

The management of dry eyes must also be guided by the objective and subjective severity of keratoconjunctivitis sicca (KCS), resulting from damage to corneal and conjunctival epithelium secondary to accelerated tear-film break-up and hyperosmolar tear composition. EULAR guidelines propose an algorithmic approach based on Ocular Staining Score (OSS) score and Ocular Surface Disease Index (OSDI) questionnaire to classify patients as non-severe or severe KCS [210]. The British Society of Rheumatology recommended a classification into 3 categories (mild, moderate and severe dry eyes) based on the Schirmer’s test, Break Up Time (BUT) and ocular staining [319]. First line therapy for all patients with dry eyes is the instillation of preservative-free artificial tears containing methylcellulose or hyaluronate, and ointment at night. In DREAM studies, use of supplements of n-3 fatty acids for 12 month and beyond does not improve OSDI, staining scores, BUT or Schirmer test compared to olive oil in dry eyes patients [323,324]. Although these treatments are not associated with an improvement in objective parameters, substantial subjective improvement in both groups suggests that daily olive oil teaspoon should be used in dry eye management [325]. Although the origin of the dryness is the decrease in the production of tears, a dysfunction of the Meibomian glands can also be associated and must be treated by daily eyelid massage with hot pad or liposomal spray to reconstitute the lipid layer preventing the evaporation of the tear film. In patients with persistent Meibomian inflammation and blepharitis, doxycycline 50 mg once daily for a minimum of 3 months is effective as a metallomatrix proteinase inhibitor. In case of refractory case of severe KCS, local treatment using NSAID-, glucocorticoid- or cyclosporin-containing eyedrops can be used under the strict supervision of an ophthalmologist. Rescue therapies by serum eye drops, oral or topical muscarinic agonists, lifitegrast-containing eyedrops or lacrimal plugs insertion must be evaluated in specialized settings.

Only two Disease Modifying Anti-Rheumatic Drug (DMARDs) have demonstrated a significant effect on sicca syndrome: Methotrexate in a small uncontrolled trial [326], and Mizoribine (a Japanese DMARD) in 2 cohort studies [327,328]. With regard to biological therapies, infliximab, etanercept, belimumab and tocilizumab have failed to demonstrate a favourable effect on exocrine glandular function in their respective RCTs. “Abatacept Sjögren Active Patients” (ASAP) proof-of-concept trial on abatacept showed a significant improvement in ESSPRI and BUT, but not on SWSF while another trial showed no effect on ESSPRI and SWSF. Some randomized trials, but not all, find an improvement in exocrine function and dryness with rituximab. In TEARS study, a study using 120 patients, aims for a >30% improvement in at least 2 VAS in 4 (fatigue, pain, dryness and PGA) at 6–16–24 weeks, primary endpoint is only reached at week 6, and this effect is no longer found thereafter. Dryness VAS is statistically different from the placebo group from week 6 to 24, but no group achieved a clinically significant decrease. The other large trial, TRACTISS, studying the effect of rituximab on 133 patients with a primary endpoint of >30% improvement oral dryness and fatigue VAS at 48w, did not show significant improvements in any outcome measure, except unstimulated salivary flow. However, this intervention does not seem cost-effective. The clinical significance of those differences remains to be determined and is interpreted according to the various guidelines. Only the Sjögren’s Syndrome Foundation proposes to use rituximab as rescue therapy for sicca syndrome [322].

In pSS patients, complaints regarding general non-specific symptoms (non-inflammatory musculoskeletal pain and fatigue) mimicking a fibromyalgia picture are common and can be challenging for the clinician. In this context, differential diagnosis is important. Non-specific manifestation of another condition (e.g., hypothyroidism, hypocortisolism, osteoarthritis, depression, neoplasia) or resulting from a misleading manifestation linked to the systemic activity of the disease (e.g., myositis, inflammatory arthralgia or arthritis, hypokalaemia or osteomalacia due to tubular involvement, small fibre neuropathy or lymphoma) must be ruled out. When no secondary cause is identified, this fibromyalgia-like presentation can be treated as such [329]. These can be quantified and monitored using the ESSPRI score or standardized scores such as the Profile of Fatigue and the Brief Pain Inventory. Education and management according to the biopsychosocial model of chronic pain, lifestyle adaptation, sleep management strategies and the practice of moderate physical activity are the cornerstones of the management of fatigue and pain. Many patients report benefit from joining a SS support group. If drug treatment is necessary, it will consist of the prescription of conventional painkillers (short-term acetaminophen or NSAID). Antidepressants and anticonvulsants may be considered as co-analgesic medications in chronic musculoskeletal or neuropathic pain, keeping in mind the anticholinergic effect of these drugs, which can worsen sicca syndrome. Opioids are not suitable treatments for chronic pain patients. DHEA supplementation is not recommended.

As a rule of thumb, systemic immunomodulatory drugs should not be used to treat non-specific systemic manifestations because evidence is scarce. In currently available biotherapies, abatacept and belimumab failed to demonstrate an effect on fatigue and pain VAS. Data on rituximab are conflicting: 3 RCTs showed an improvement in fatigue VAS, results not found in the large TRACTISS trial. A phase 2 RCT on a total of 17 patients failed to demonstrate >20% improvement of fatigue VAS at 24 weeks, fatigue VAS improvement at 24w or >30% improvement of fatigue VAS at 24w. The authors only report a statistically significant improvement in fatigue VAS in treated group compared to baseline, while the placebo group did not reach a statistically significant difference [330]. In two other studies, patients with early pSS and active disease treated with RTX displayed a significant improvement in fatigue VAS compared to placebo from different time points post-treatment [331,332]. All RCTs have shown that rituximab is not associated with an improvement in pain VAS. An RCT investigating the effect of anakinra on fatigue, although not reaching its primary endpoint, shows a significant improvement in VAS fatigue [333]. Off-label use of DMARD or biological treatments, even as a rescue therapy, is currently not mainstream recommendation in this indication. However, some guidelines suggest a trial of hydroxychloroquine in patients with recurrent musculoskeletal complaints or fatigue, mainly based on “experience-based medicine”. In its 2015 guidelines, Sjögren’s Syndrome Committee of Brazilian Society of Rheumatology highlighted the possibility of using rituximab as rescue therapy for fatigue (but not sicca syndrome) management [320].

### 7.2. Systemic Manifestations

Management of visceral manifestations linked to disease systemic activity is currently based only on rare randomized controlled trials, cohort studies or case-reports [334]. Treatment regimens are often borrowed from systemic lupus erythematosus (SLE), rheumatoid arthritis (RA), mixed cryoglobulinemia or idiopathic organ-specific autoimmune disease management.

Therapeutic regimen must be tailored to organ specific involvement and severity of the disorder. This approach requires organ-by-organ examination of disease activity and pre-existing damage. To this end, ESSDAI score may be used as a guide but does not take into count all the systemic manifestations of pSS [210]. As a rule of thumb, systemic immunosuppressive therapy will only be offered to patients with moderate or severe organ activity (as define in ESSDAI score) or moderate overall systemic activity (ESSDAI ≥5) [210]. Organ manifestation classified as mild usually requires only self-care advice, local treatment or pain relief medication (NSAID for inflammatory arthralgia or co-analgesic for neuropathic pain). In case of treatment failure, low-dose corticosteroid treatment and/or conventional DMARD may be considered depending on clinical manifestation.

In cases requiring immunosuppressive therapy, an induction/remission biphasic regimen is recommended for the rapid control of organ damage and the preservation of its function [210]. Corticosteroid therapy is an almost essential treatment for moderate to severe systemic manifestations. To date, no steroid-free regimen has been studied in pSS and 95% of the published regimens include corticosteroid therapy, alone or in combination with an immunosuppressant [210]. When immunosuppressive therapy is prescribed, it is usually a conventional broad-spectrum immunosuppressant used as a cortisone-sparing or as a remission-inducing agent: hydroxychloroquine, methotrexate, other conventional DMARDs (leflunomide, salazopirine), mycophenolate mofetil or cyclosporine. As there are no head-to-head comparisons, the choice of immunosuppressant is mainly based on the clinician’s experience and on the therapeutic regimens used in idiopathic or lupus-related disorders (HCQ and MTX in skin and articular involvement, AZA, CyA or MMF in pulmonary or renal involvement). Severe life- or organ-threatening manifestations (central nervous system involvement, glomerulonephritis), generally require an aggressive regimen including methylprednisolone pulse-therapy combined with an alkylating agent (usually cyclophosphamide IV or PO, more rarely chlorambucil) as remission-inducing agents. IVIG at immunomodulatory doses are used in neuropathies or myositis. Biological therapies (mainly rituximab) generally come only in the third line as rescue therapies. The exception to this rule concerns the manifestations associated with cryoglobulinemia where rituximab is proposed as an immunosuppressant of choice, in combination with corticosteroid therapy or even plasmapheresis in life-threatening cases. As with other autoimmune diseases, corticosteroid therapy should be reasoned with a tapering regimen guaranteeing the shortest possible exposure to supraphysiological doses while maintaining remission. Complications of chronic corticosteroid therapy must be addressed proactively.

Hydroxychloroquine is commonly used as first line DMARD for moderate systemic manifestations mainly affecting the skin and joints. Its use is mainly based on the similarities between pSS and SLE, as pSS is sometimes considered as “lupus of mucous membranes”. As opposed to SLE, the evidence for its use in systemic manifestations of pSS does not actually exist, and its use is completely empirical. The first—JOQUER trial—attempting to demonstrate the effect of hydroxychloroquine over 24 weeks failed to reach the primary endpoint (30% or greater reduction between weeks 0 and 24 in scores on 2 of 3 VAS (dryness, pain, and fatigue)) [335]. In a more recent RCT performed over 2 weeks, no effect of hydroxychloroquine was seen on BUT test, Schirmer test, corneal staining score or OSDI score [336]. While those RCT have not been designed to investigate the effect of the drug on systemic manifestations of the disease, and the number of patients was small, hypergammaglobulinemia statistically improved significantly [335,336].

### 7.3. pSS-Associated Lymphoma

The occurrence of lymphoma is a complication that must be screened clinically, especially in patients at risk (see above). Any appearance of a firm, painless glandular swelling must be investigated if it does not disappear spontaneously. The exams of choice to detect lymphoma are an MRI of the major SG and a CT of chest, abdomen and pelvis for staging or a PET scan to investigate the entire body in a single examination. pSS patients with lymphoma require personalized treatment provided by an oncohematologist according to the histological type, the extent of the involvement and the systemic manifestations.

### 7.4. Obstetrical Considerations

Ideally, pSS patients of childbearing age should benefit from a preconception consultation aimed at reviewing their treatment and their serological profile (anti-Ro/SSA, anti-La/SSB and antiphospholipid panel). Low-dose aspirin can be considered to promote placental implantation [319]. Anti-Ro/SSA positive mothers should be followed regularly by foetal ultrasound in a specialized centre [210,319]. Prophylactic treatment of neonatal atrioventricular block with hydroxychloroquine may be offered, since this drug is compatible with pregnancy [210]. If a conduction disorder appears on a follow-up ultrasound, rescue therapy with glucocorticoid with or without IVIG may be attempted [210]. In the event of atrioventricular block at birth, a pacemaker must be quickly implanted.

### 7.5. Targeted Therapies: Revolution or Disillusion?

**Figure 4:**
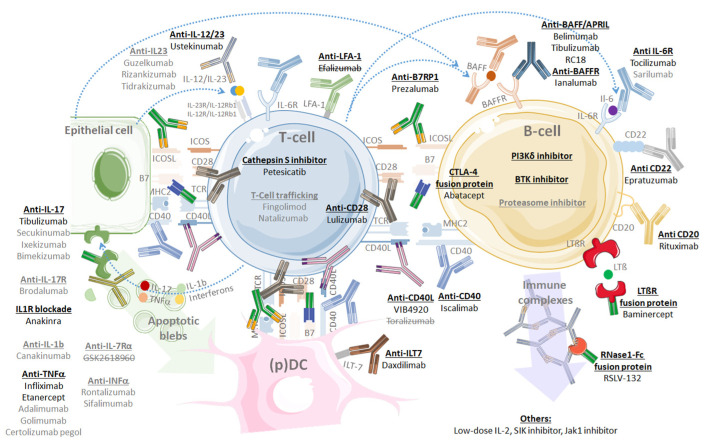
Synoptic view of targeted drugs (being) studied in pSS. Therapeutic classes are in bold. Biotherapies and small molecules are in black if they have been the subject of one or more trials in pSS or in grey if they exist but have not been tested in pSS. Names in strikethrough are drugs whose development has been stopped because of unacceptable side effects or because of portfolio prioritization.

Targeted therapies have revolutionized Rheumatology in recent years, especially in chronic inflammatory rheumatism—such as in RA—and, to a lesser extent, systemic diseases such as SLE and vasculitis. In terms of pSS, many targeted therapies have been tested or are currently in the pipeline. Unfortunately, a revolution like the one known in the field of RA has not yet occurred. These targeted drugs are shown in Figure 4 and summarized in Table 6, Table 7, Table 8, Table 9, Table 10, Table 11 and Table 12.

Given their predominant role in the production of autoantibodies, germinal centres and the evolution towards lymphoma, B cell depletion is one of the therapeutic mechanisms studied in pSS (Table 6, Table 7 and Table 8). In addition to the mixed results of the anti-CD20 Rituximab RCTs, other targeted drugs have been studied. Epratuzumab, an anti-CD22 B cell depleting therapy studied in SLE patients had a positive effect on the systemic activity of SLE patients with Sjögren syndrome in a post-hoc analysis of EMBODY trial [337]. However, an RCT should be designed to assess the effect of the therapy on both ESSDAI and ESSPRI in pSS patients. Other B cell depletion strategies aiming at blocking the BAFF pathway showed a positive effect on the ESSDAI and ESSPRI scores at 28–52 weeks [338,339]. However, the confirmation of these promising results against a placebo is necessary. Other strategies targeting BAFF pathway are also under investigation: a TACI-antibody fusion protein called RC18, rituximab + belimumab combo therapy, Tibulizumab—a dual anti-BAFF (belimumab) and anti-IL-17 antibody (Ixekizumab)—and Ianalumab (anti-BAFF receptor). The results of these different studies are expected during 2020. B cell targeting drugs by Bruton tyrosine kinase inhibitor (4 molecules), LTßR fusion protein, PI3Kδ inhibitor (3 molecules) and Cathepsin S inhibitor are currently being evaluated with inconclusive results to date. Bortezomib, a proteasome inhibitor used for the treatment of multiple myeloma, has been successfully used in 2 cases of refractory pSS reports but has never been studied on a larger scale [236,340].

T-cells play a central role in the modulation and polarization of the local autoimmune reaction within lymphocyte infiltrates in the exocrine glands. They are also used as therapeutic target by biotherapies interfering with the T-cell co-stimulation (Table 9). To date, there is no convincing result to recommend these treatments in pSS, but most studies targeting the CD40-ligand (CD154)/CD40 pathway are in progress. Therapies targeting T-cell trafficking, such as Fingolimod or Natalizumab, have not been studied in pSS.

With regard to anti-cytokine targeted therapies, RCTs using anti-TNF (infliximab and etanercept) and anti-IL6 receptor (tocilizumab) are negative (Table 11). Anakinra demonstrated a statistically significant decrease in fatigue VAS, without however reaching its primary clinical endpoint. The development of GSK2618960—an anti-IL-7Rα biotherapy—was stopped by the company due to the prioritization of their portfolio. So far, only one RCT studying the effect of Ustekinumab—an anti-IL-12/IL-23 antibody—on ESSDAI score at week 24 as primary endpoint is expected to give results in 2022 [341].

In a phase II trial, Filgotinib—a Jak1 inhibitor—and Lanraplenib—a SIK inhibitor—failed to demonstrate a significant effect on the ESSDAI and ESSPRI scores [342]. Finally, innovative therapies targeting plasmacytoid dendritic cells, immune complexes by RNase1-Fc fusion protein or the induction of T-reg cells by low-dose IL-2 injections are being evaluated. These various therapies are reviewed in Table 12.

## 8. Conclusions

pSS is a multifaceted disease combining pleiomorphic systemic autoimmune manifestations, glandular manifestations, a frequently added psychosomatic component and the possible progression to non-Hodgkin lymphoma. Its management has two complementary facets: improving the quality of life of patients by tackling dryness, fatigue and chronic pain symptomatically in a multidisciplinary way and treating systemic manifestations to prevent damage, which will worsen the vital and functional prognosis. Although we understand more and more its pathophysiology, many questions remain unanswered, and its treatment remains disappointing compared to other autoimmune diseases. pSS therefore remains a vast field of investigation where much fundamental and clinical research remains to be done. Ten take-home messages:

1. SS is characterized by lymphoplasmacytic infiltration of exocrine glands. The cause of SS is complex and influenced by a combination of genetic, epigenetic, hormonal and environmental factors.
2. The pathogenic mechanisms remain unclear. However, the immune system-mediated loss of glands function, specifically of salivary and lacrimal glands, certainly explains the common symptoms of dry mouth and dry eyes. In this inflammatory environment, T-cells mediate a direct destruction of glandular tissue and B-cell activation, leading to the production of autoantibodies. More than 20 autoantibodies could be involved in SS, but the most commonly used for SS diagnosis are anti-Ro/SSA and anti-La/SSB.
3. Although often reduced to its sicca syndrome due to its tropism for glandular tissue, pSS remains a systemic disease that can affect virtually all organs. These clinical manifestations can be due to various mechanisms: dryness secondary to exocrinopathy, autoimmune epithelitis with periepithelial lymphocytic infiltration of target organs, autoimmunity and clonal lymphocytic expansion.
4. Due to its protean and willingly insidious presentation, pSS is sometimes difficult to recognize and may delay diagnosis by more than 10 years. Classification criteria are used to create cohorts for study purposes and should not be used blindly as diagnostic criteria but as a guide in clinical practice. For these various reasons, the gold standard for individual diagnosis of pSS remains the opinion of an expert clinician.
5. From a serohistological point of view, so-called “secondary Sjögren’s syndrome” in SLE and SScl patients does not differ from pSS. It is therefore preferable to forget this historical dichotomy. In this way, the clinician avoids three pitfalls: (1) minimizing the SS-related symptoms, which decrease the quality of life of the patients; (2) forget that overlap may change the clinical phenotype and (3) forget the risk of lymphoma.
6. Although overall pSS mortality is low and similar to the general population, a subgroup of patients will have a poorer vital prognosis linked to cardiovascular events, solid-organ and lymphoid malignancies and infections. Biomarkers associated with the development of MALT lymphoma are mainly signs associated with exuberant B cell proliferation and immune-complex production.
7. The impact of pSS can be assessed according to three clinical dimensions: “sicca asthenia polyalgia” complex, inflammatory disease activity and structural damage. They are assessed by the ESSPRI, ESSDAI and SSD(D)I scores, respectively. Even in the absence of florid systemic manifestations, pSS can be disabling and associated with significant functional status impairment related to oral and/or ocular dryness, systemic activity, pain, fatigue and daytime somnolence, anxiety and depression symptoms.
8. The treatment of manifestations linked to the “sicca asthenia polyalgia” complex mainly involves symptomatic measures and rehabilitation. To date, no immunosuppressant has demonstrated a favourable risk–benefit balance in this indication.
9. The treatment of manifestations related to inflammatory disease activity is currently based on scarce evidence. Therapeutic regimen must be tailored to organ specific involvement and severity of the disorder. Mild manifestations will be treated with hydroxychloroquine or local corticosteroids while moderate to severe systemic involvement will require the use of systemic corticosteroid therapy, combined or not with a broad-spectrum immunosuppressant. Rituximab will only be used as a third line, except in cases of cryoglobulinemia where it is the treatment of choice.
10. Despite targeted therapies having revolutionized rheumatology in recent years and the impressive number of molecules tested so far in pSS, a revolution like the one known in the field of RA has not yet occurred.

## Figures and Tables

**Table 1:** Rapid overview of original publications describing novel autoantibodies in pSS.

Autoantigen Targeted by Autoantibody	Number of Patients (N Total/Pooled)					Autoantibody Prevalence (% of Total)					Clinical Associations
	pSS	pSS MALT	Sicca	FM Sicca	Crtl	pSS	pSS MALT	Sicca	FM Sicca	Crtl	
Salivary protein 1 (SP1)	270	_	29	151	148	46.3	_	75.9	45.7	27	Early disease, low focus-score, SSA−/SSB− [169,170,171,172,173] Found in non-pSS dry eye and fibromyalgia with sicca syndrome [167,174,175]
Carbonic anhydrase 6 (CA6)	13	_	_	151	23	53.8	_	_	7.3	4.3	
Parotid secretory protein (PSP)	13	_	_	151	23	15.4	_	_	11.3	4.3	
Interferon-inducible protein-16	250	_	_	_	255	37.2	_	_	_	2.7	High focus-score and GC, hyperγ, ANA > 1:320 [176]
Mouse double minute 2 (MDM2)	100	_	_	_	74	21	_	_	_	5.4	⇧ disease duration, ESSDAI, ⇧ focus-score, anaemia, thrombocytopenia, SSB+ [177]
Nuclear autoantigen 14 kDa (NA-14)	204	_	_	_	144	12.7	_	_	_	0	⇧ IgA level, ANA < 1:320, ANA−, shorter disease duration [178,179]
Stathmin-4	72	_	_	_	128	15	_	_	_	5	Polyneuropathy, vasculitis [180]
Poly(U)-binding splicing factor 60 kDa	84	_	_	_	38	30	_	_	_	5.3	Asian or African descent, ANA+, RF+, hyperγ, SSA+, SSB+ [181]
NR2	66	_	_	_	99	20	_	_	_	7.6	⇩ memory function, ⇧ depression rate [182] ⇩ hippocampal grey matter [183]
	50	_	_	_	_	12 *	_	_	_	_	
TRIM38	235	_	_	_	50	10	_	_	_	4	⇧ ocular stain scores, ⇩ Schirmer’s test, focus-score ≥ 3, SSA+, RF+, hyperγ [184]
Saccharomyces cerevisiae	104	_	_	_	_	5	_	_	_	_	Triple Ro52+/Ro60+/La+, hypocomplementemia, cutaneous involvement [185]
Calponin-3	209	_	_	_	46	11	_	_	_	2.2	Peripheral neuropathy [186]
Ganglionic acetylcholine receptor	39	_	_	_	39	23	_	_	_	0	Autonomic neuropathy [187]
Aquaporin-4	109	_	_	_	_	10	_	_	_	_	NMOSD overlap [188]
Aquaporin-5	112	_	_	_	53	73	_	_	_	32	Low resting salivary flow [164]
Other aquaporins (1, 3, 8, 9)	34	_	_	_	_	38	_	_	_	_	⇧ ocular stain scores [189]
P-selectin	70	_	_	_	35	21		_	_	0	Low platelet count [190]
Carbamylated proteins	123	_	_	_	172	28.5	_	_	_	3.5	⇧ total IgG, IgM, RF+, β2-microglobulin, ⇧ focus-score and GC [191,192]
Moesin	50	_	_	_	50	42	_	_	_	4	[193]
Cofilin-1	50	20	_	_	50	76	80	_	_	18	Association with pSS lymphoma [194] IgA isotype of anti-Ro/SSA ACPA+ and high urine pH for anti-alpha-enolase [195]
Alpha-enolase	50	20	_	_	50	82	90	_	_	26	
Rho GDP-dissociation inhibitor 2	50	20	_	_	50	86	90	_	_	26	

* = antibody positivity in cerebrospinal fluid; Sicca = non-pSS Sicca syndrome; FM = fibromyalgia with non-pSS sicca syndrome similar to “Sicca Asthenia Polyalgia” syndrome; Crtl = healthy controls; hyperγ = hypergammaglobulinemia; ANA = antinuclear antibodies; GC = germinal centre; SSA and SSB = anti-Ro/SSA (Ro52 and/or Ro60) and anti-La/SSB; ESSDAI = Eular Sjögren Syndrome Disease Activity Index; RF+ = rheumatoid factor positivity; NMOSD = Neuromyelitis Optica Spectrum Disorder; ACPA+ = anti-citrullinated protein antibodies positivity; ⇧ = increase(d)/higher; ⇩ = decrease(d)/lower; “−” = negativity.

**Table 2:** Modern pSS Classification Criteria—comparisons of items, definitions and diagnosis performance compared to experts’ opinions.

	AECG Classification Criteria (2002) [250]		SICCA Classification Criteria (2012) [251]		ACR-EULAR Classification Criteria (2016) [252]	
Domain	Item Definition	Value	Item Definition	Value	Item Definition	Value
Subjective eye dryness	≥1/3 specific questions	minor	/	_	/	_
Subjective oral dryness	≥1/3 specific questions	minor	/	_	/	_
Ocular signs	Schirmer (≤5 mm/5 min) OR Van Bijsterveld ≥ 4	minor	OSS ≥3	1	Schirmer (<5 mm/5 min)	1
					OSS ≥ 5 OR Van Bijsterveld ≥ 4	1
SG dysfunction	UWSF (≤1.5 mL/15 min) OR Compatible parotid sialography OR Anormal salivary scintigraphy	minor	/	_	UWSF (≤0.1 mL/min)	1
MSGB	Focus-score ≥ 1	Major	Focus-score ≥ 1	1	Focus-score ≥ 1	3
Auto antibodies	Anti-Ro/SSA or Anti-La/SSB	Major	Anti-Ro/SSA or Anti-La/SSB OR RF(+) with ANA(+) ≥1:320	1	Anti-Ro/SSA	3
pSS definition	4 out of 6 with ≥ 1 Major (or 3 out of 4 objectives findings)		pSS signs and/or symptoms with ≥2/3 criteria		Sicca or ESSDAI manifestation with a total score ≥ 4	
Exclusions criteria	- Past head and neck radiation - Hepatitis C infection - AIDS - Pre-existing lymphoma - Sarcoidosis - Graft-versus-host disease - Current use of anticholinergic drugs		- Past head and neck radiation - Hepatitis C infection - AIDS - Sarcoidosis - Graft-versus-host disease		- Past head and neck radiation - Hepatitis C infection - AIDS - Pre-existing lymphoma - Sarcoidosis - Graft-versus-host disease - Amyloidosis - IgG4-related disease - Current use of anticholinergic drugs	
Sensitivity	93.5%		92.5%		96%	
Specificity	94.0%		95.4%		95%	

AECG = American European Consensus Group, SICCA = Sjögren’s International Collaborative Clinical Alliance, ACR-EULAR = American College of Rheumatology—European League Against Rheumatism, UWSF = unstimulated whole saliva flow, RF = rheumatoid factor, ANA = antinuclear antibodies, ESSDAI = EULAR Sjögren’s syndrome disease activity index.

**Table 3:** Differential diagnosis of Sjögren’s syndrome (non-exhaustive list).

	Sicca Symptoms Complex	Glandular Involvement	Articular Involvement	Systemic Involvement
Xerogenic medications	X	_	_	_
Aromatase inhibitors	(X)	_	X	(X) pSS-like
Age-related dryness	X	_	_	_
Metabolic sialadenosis	_	X	_	_
Non-SS dry eye diseases	X	_	_	_
Head and neck irradiation	X	_	_	_
Sarcoïdosis	X	X	X	X
Hyperlipoproteinemia (II, IV, V type)	X	X	(X)	_
Chronic Graft vs. Host disease	X	X	X	X
Primary lymphoma	X	X	_	(X)
Amyloïdosis	X	X	(X)	(X) Renal, purpura
Viral chronic sialadenitis (HCV, HIV, HTLV-1)	X	(X)	X	X
Other chronic Non-specific sialadenitis	X	X Usually unilateral	_	_
Diabetes Mellitus	X	(X) Sialadenosis	(X) Cheiroarthropathy	(X) Neuropathy
Haemochromatosis	X	(X)	X CPPD	(X)
Other connective tissue disease	X	_	X	X
Rheumatoid arthritis	(X)	_	X	(X)
Granulomatosis with polyangiitis	X	(X)	X	X
IgG4-related disease (Mikulicz syndrome)	X	X	(X)	(X)
Anxiety, fibromyalgia	X	_	(X)	_
Checkpoint inhibitors	X	(X)	X	X

**Table 4:** Common damage, burden and activity scores for clinical monitoring of pSS patients.

	EULAR Sjögren’s Syndrome Disease Activity Index	EULAR Sjögren’s Syndrome Patient Reported Index	Sjögren’s Syndrome Disease Damage Index	Sjögren’s Syndrome Damage Index
**Abbreviation**	ESSDAI	ESSPRI	SSDDI	SSDI
**First description**	Seror et al. [294]	Seror et al. [295]	Vitali et al. [296]	Barry et al. [297]
**Year**	2010	2011	2007	2008
**Type**	Activity index	PRO	Damage index	Damage index
**Domains (n)**	12	1	6	9
**Items (n)**	44	3	9	27
**Items scoring**	0 to 3	VAS (0–10)	1, 2 or 5	1
**Domain weight**	1 to 6	1	1	1
**Calculation**	Sum	Mean	Sum	Sum
**Score range**	0–123	0–10	0–16	0–27
**Clinically significant** **threshold**	<5 Low ≥5, ≤13 moderate ≥14 high	≥5/10 is an unsatisfactory symptom state	-	-
**Minimal clinically important difference**	≥3 points improvement	≥1 point or ≥15% improvement	-	-

VAS = visual analogue scale, PRO = patient reported outcome.

**Table 5:** Current treatment for sicca-related manifestations.

	Salivary Gland Involvement	Lachrymal Gland Involvement	Skin and Vaginal Mucosa Involvement
Self-Care	- Environment humidification - Elimination of offending drugs - Avoidance of caffeine, alcohol - Avoidance of tobacco - Excellent oral hygiene - Limit acidic and sugar intake - Limit eating between meals - Chew xylitol-containing gum	- Environment humidification - Elimination of offending drugs - Excellent ocular hygiene	
Conserve		- Scleral contact lenses	
Replace	- Salivary substitutes	- Artificial tears - Liposomal spray - Autologous serum drops	- Vaginal lubricants - Topical oestrogen
Stimulate	- Mechanical stimulants (gums) - Pilocarpine PO - Pilocarpine mouthwash - Cevimeline PO - Choleretic (anetholtrithione) - Mucolytic (NAC, bromhexine) - Electrostimulation	- Pilocarpine 5 mg q6h PO - Pilocarpine eye drops - Cevimeline 30 mg q8h - Lid hygiene with hot pad - Diquafosol eye drops (Japan) - Rebamipide eye drops (Japan)	- Pilocarpine 5 mg q6h PO - Cevimeline 30 mg q8h
Complications Prevention and Management	- Fluoride mouthwash - Chlorhexidine mouth bath In case of candida infection - Oral nystatin - Fluco-/Itraconazole In case of glands swelling - Exclude stone or infection - Massaging major glands	- NSAID or glucocorticoid drops - Calcineurin inhibitors drops - Lifitegrast eye drops - Botulinium toxin treatment - Corneal grafting - Doxycycline PO	

**Table 6:** B cell targeted drugs in pSS part 1: monoclonal antibodies directed against B cell specific Cluster of Differentiation (CD).

DRUG	TRIAL (Reference)	Inclusion Criteria	Number of Subjects		Age (Years)	Disease Duration (Years)	Mean ESSDAI	Primary Outcome	Results	Effects (Statistically Significant)		
			Drg	Ctrl						Sicca Syndrome	Fibro-Like	Systemic
**Rituximab Anti-CD20**	NCT00363350 Phase I/II [343]	AECG criteria RF+ and SSa and/or SSb+ SWS >0.15 mL/min	20	10	43 ± 11	5.25 ± 4.17	8 (4–13)	SWS ⇧ at 48w	met	SWS/UWS ⇧ LG test ⇧ Schirmer = BUT =	SF36 ⇧ MFI ⇩	Vasculitis ⇩
	Phase III [330]	AECG criteria SSa and/or SSb+ F-VAS ≥5/10	8	9	51 (22–64)	7.25 (1–18)	na	⇩ > 20% of F-VAS at 24w; ⇩ F-VAS at 24w; ⇩ >30% of F-VAS at 24w	not met not met not met	UWS = Schirmer =	F-VAS ⇩ PROFAD ⇩ P-VAS = Soc-SF36 ⇧	Glandular ⇩
	Phase III [332]	AECG criteria SSa and/or SSb+ Disease duration ≤ 2y 2/5 of [PhGA >50 mm or ESSDAI ≥ 6 or subESSPRI ≥ 5]	19	22	40 (27–53)	1 (1–2)	20 (6–41)	ΔESSDAI until 120W	met from 24w to 120w	D-VAS ⇩ Schirmer ⇧ UWS ⇧	P-VAS = F-VAS ⇩	ESSDAI ⇩
	NCT00740948 Phase III TEARS [331]	AECG criteria with 2/4 VAS ≥ 5/10 for PhGA, pain, fatigue and dryness AND biologically active OR 1 extra-glandular manifestation or parotid gland enlargement.	63	57	52.9 ± 13.3	4.6 ± 4.8	10 ± 6.9	⇩ 30% of at least 2/4 VAS at 6-16-24w	met at 6w not met at 16-24w	D-VAS ⇩ Schirmer =	P-VAS = F-VAS ⇩	ESSDAI = Glandular = Articular =
	Phase III TRACTISS [344]	pSS with SSa+ UWS >0 mL/min F-VAS and D-VAS >5/10	67	66	54 ± 11.5	5.7 ± 5.4	5.7 ± 4.5	⇩ 30% D-VAS and F-VAS at 48w	not met	UWS ⇧ ESSPRI = D-VAS =	F-VAS = SF36 = PROFAD =	ESSDAI ⇩
**Epratuzumab Anti-CD22**	Post-hoc Phase I/II EMBODY [337]	SLE with SSa+ and SS diagnosis	31 + 41	40	46.4 ± 12.3	5.1 (0–34)	na	BICLA at 48w ΔBILAG at 48w ΔSLEDAI at 48w ΔPhGA at 48w	met met not met not met	na	na	BILAG ⇩

AECG = American European Consensus Group, Drg = drug/treatment group, Ctrl = control group, Fibro-like = fibromyalgia-like symptoms such as fatigue and widespread pain, FR+ = presence of rheumatoid factor, SSa/SSb = anti-Ro/SSa and anti-La/SSb, SWS = stimulated whole saliva flow, UWS = unstimulated whole saliva flow, LG test = lissamine green test, BUT = break-up time, SF36 = Short Form 36 health survey score, Soc-SF36 = social component of SF36 score, Phys-SF36 = physical component of SF36 score, MFI = Multidimensional Fatigue Inventory score, F-VAS = fatigue visual analogue scale, Schirmer = Schirmer test, P-VAS = Pain visual analogue scale, PROFAD = Profile of Fatigue and Discomfort, DSST = Digit Symbol Substitution Test, ESSDAI = EULAR SS disease activity index, D-VAS = dryness visual analogue scale, PhGA = physician global activity visual analogue scale, subESSPRI = P-VAS, D-VAS or F-VAS, BILAG = British Isles Lupus Assessment Group index, BICLA = BILAG-based Combined Lupus Assessment, ESSPRI = EULAR SS Patient Reported Index, SAEs = serious adverse effects, SGUS = salivary gland ultrasound, Ig = immunoglobulin, ⇩⇧ = decrease/increase, Δ = difference.

**Table 7:** B cell targeted drugs in pSS part 2: BAFF/APRIL system targeted therapies.

DRUG	TRIAL (References)	Inclusion Criteria	Number of Subjects		Age (Years)	Disease Duration (Years)	Mean ESSDAI	Primary Outcome	Results	Effects (Statistically Significant)		
			Drg	Ctrl						Sicca Syndrome	Fibro-Like	Systemic
**Belimumab Anti-BAFF**	NCT01160666 NCT01008982 Phase II BELISS [338,339]	AECG criteria SSa and/or SSb+ AND systemic complication OR B cell activation OR early disease (≤5 years)	30	-	49.5 ± 6.5	5.7 ± 5.6	8.8 ± 7.4	⇩ of 2/5 VAS at 28w - ≥ 30% D-VAS; - ≥ 30% F-VAS; - ≥ 30% P-VAS; - ≥ 30% PhGA; - ≥ 25% B cell markers	60% response	ESSPRI ⇩ D-VAS ⇩ UWS = Schirmer =	ESSPRI ⇩ P-VAS = F-VAS = SF36 =	ESSDAI ⇩ Glandular ⇩
		Follow-up of previous study	15	-	40.2 ± 11.8	5.9 ± 5.7	3.8 ± 3.1	Idem between 28–52w	86.7% Stable response	ESSPRI ⇩ D-VAS ⇩ UWS = Schirmer =	ESSPRI ⇩ P-VAS = F-VAS = Phys-SF36⇧	ESSDAI ⇩ Glandular ⇩ Articular ⇩ Biologic ⇩
**RC18** **TACI-Igfusion protein**	NCT04078386 Phase II [345]	AECG criteria SSa+ ESSDAI ≥ 5	30		?	?	?	ΔESSDAI at 24w	December 2020	Secondary endpoint	Secondary endpoint	Primary endpoint
**Rituximab** **Anti-CD20** **+** **Belimumab** **Anti-BAFF**	NCT02631538 Phase II [346]	AECG criteria SSa and/or SSb+ ESSDAI ≥ 5 UWS >0 mL/min D-VAS ≥ 5/10	70		?	?	?	SAEs at 104w AESIs at 104w	Study completed on June 2020	Secondary endpoint	na	Secondary endpoint
**Tibulizumab (LY3090106)** **Anti-BAFF +** **Anti-IL-17**	NCT02614716 Phase I [347]	AECG criteria SSa and/or SSb+	32		?	?	?	SAEs at 197d	Not published	na	na	na
**Ianalumab** **(VAY736)** **Anti-BAFFR**	NCT02149420 Phase II [348]	AECG criteria ANA ≥1:160 SSa and/or SSb+ ESSDAI ≥ 6 UWS >0 mL/min	6+12	9	50.5 ± 12.16	?	12.5 (6, 31)	ΔESSDAI at 12w	not met	D-VAS ⇩	SF-36 = MFI ⇩ F-VAS ⇩	ESSDAI = Articular ⇩
	NCT02962895 Phase II [349]	AECG criteria SSa+ ESSDAI ≥ 6 (from 7 domains only)	195		?	?	?	Change in multi-dimensional disease activity at 24w	Study completed on June 2020	Secondary endpoint	Secondary endpoint	Primary endpoint

**Table 8:** B cell targeted drugs in pSS part 3: drugs targeting other B cells survival and function pathways.

DRUG	TRIAL (Reference)	Inclusion Criteria	Number of Subjects		Age (Years)	Disease Duration (Years)	Mean ESSDAI	Primary Outcome	Results	Effects (Statistically Significant)		
			Drg	Ctrl						Sicca Syndrome	Fibro-Like	Systemic
**LOU064** **BTK inhibitor**	NCT04035668 Phase II LOUISSe [350]	2016 ACR/EULAR criteria SSa and/or SSb+ ESSDAI ≥ 6 UWS >0 mL/min	252		?	?	?	ΔESSDAI at 24w	Estimated Study Completion on January 2023	Secondary endpoint	Secondary endpoint	Secondary endpoint
**Tirabrutinib** **(GS-4059)** **BTK inhibitor**	NCT03100942 Phase II [342]	AECG criteria SSa and/or SSb+ ESSDAI ≥ 4	38	37	55.8 ± 10.06	?	10.4 ± 5.36	Protocol-Specified Response Criteria at 12w	not met	ESSPRI =	ESSPRI =	ESSDAI =
**BMS-986142** **BTK inhibitor**	NCT02843659 Phase II [351]	2016 ACR/EULAR criteria SSa and/or SSb+ ESSDAI ≥ 6 UWS >0 mL/min	5+6	7	51.2 ± 11.41	?	?	ΔESSDAI at 12w	Not published	Secondary endpoint	Secondary endpoint	Secondary endpoint
**Branebrutinib** **BTK inhibitor**	NCT04186871 Phase II [352]	2016 ACR/EULAR criteria Moderate to severe pSS	?	?	?	?	?	Protocol-Specified Response Criteria at 24w	Estimated Study Completion on June 2022	na	na	Primary endpoint
**Baminercept** **LT** **β** **-R** **fusion protein**	NCT01552681 Phase II [353]	2016 ACR/EULAR criteria UWS >0.1 mL/min ≥ 1 non-life-threatening systemic manifestation(s)	33	19	52.0 ± 11.0	?	3.1 ± 3.4	ΔSWS at 24w	not met	D-VAS = Schirmer ⇧ UWS =	F-VAS =	ESSDAI =
**Parsaclisib (INCB050465)** **PI3Kδ inhibitor**	NCT03627065 Phase II [354]	AECG criteria SGUS score > 2 SSa and/or SSb+ ESSDAI ≥ 6 Oral dryness score ≥ 5.	10		?	?	?	ΔSGUS score at 12w	Not published	na	na	na
**Seletalisib** **(UCB5857)** **PI3Kδ inhibitor**	NCT02610543 Phase II [355]	AECG criteria FAN ≥ 1:160 SSa and/or SSb+ ESSDAI ≥ 6	13	14	?	?	?	ΔESSDAI at 12w	not met	ESSPRI = SWSF = Schirmer =	na	ESSDAI =
**Leniolisib** **(CDZ173)** **PI3Kδ inhibitor**	NCT02775916 Phase II [356]	pSS diagnosis SSa and/or SSb+ ESSDAI ≥ 6, ESSPRI ≥ 5 SWS > 0.1 mL/min	20	10	47.3 ± 13.07	?	?	ΔESSDAI at 12w SAEs at 12w	not met	ESSPRI =	SF-36 = MFI =	na

**Table 9:** T-cell targeted drugs in pSS: co-stimulation receptors or ligands inhibition.

DRUG	TRIAL	Inclusion Criteria	Number of Subjects		Age (Years)	Disease Duration (Years)	Mean ESSDAI	Primary Outcome	Results	Effects (Statistically Significant)		
			Drg	Ctrl						Sicca Syndrome	Fibro-Like	Systemic
**Abatacept** **CTLA-4 Ig** **fusion protein**	NCT02915159 Phase III [357]	2016 ACR/EULAR criteria SSa+ ESSDAI ≥ 5	92	95	52 ± 12.9	**?**	9.4 ± 4.3	ΔESSDAI at 169d	Not met	ESSPRI = SWS =	ESSPRI =	ESSDAI = DAS28 ⇩
	Phase I/II ASAP [358]	AECG criteria and ESSDAI ≥ 6 Disease duration ≤ 5 years SWS ≥ 0.10 mL/min SSa and/or SSb+ or FR+ Proven by parotid gland biopsy.	15	-	43 (32–51)	11 (7–36)	11 (8–14)	ΔESSDAI at 24-48w	met	ESSPRI ⇩ SWS/UWS = Schirmer = BUT ⇧	ESSPRI ⇩	ESSDAI ⇩
	NCT02067910 Phase III ASAPIII [359]	AECG criteria and ESSDAI ≥ 5 Time from diagnosis ≤ 7 years	40	39	49 ± 16	8 (4–14)	?	ΔESSDAI at 24w	Not met	ESSPRI ⇩ FSFI ⇧ DVAS = UWS = Schirmer =	Fatigue =	ESSDAI = Articular ⇩
**Iscalimab (CFZ533)** **Anti-CD40**	NCT02291029 Phase IIa [360]	AECG criteria and ESSDAI ≥ 6 SSA+ OR FR+ and FAN ≥ 1:320 SWS ≥ 0 mL/min	8+21	4+11	51.3 ± 13.5	?	10.7 ± 4.6	SAEs at 12w	safe	ESSPRI ⇩ UWS = Schirmer =	MFI = SF-36 =	ESSDAI ⇩ Articular ⇩
	NCT03905525 Phase II TWINSS [361]	2016 ACR/EULAR criteria SSa+ SWS > 0.01 mL/min, P1: ESSDAI ≥ 5 or P2 ESSPRI ≥ 5.	260		?	?	?	ΔESSDAI at 24w in P1 ΔESSPRI at 24w in P2	Estimated Study Completion on June 2022	Included endpoint	Included endpoint	Included endpoint
**VIB4920** **MEDI4920** **Anti-CD40L**	NCT04129164 Phase II [362]	P1: ESSDAI ≥ 5 P2: ESSDAI < 5 et ESSPRI ≥ 5	174		?	?	?	ΔESSDAI at 169d in P1 ΔESSPRI at 169d in P2	Estimated Study Completion on April 2022	Included endpoint	Included endpoint	Included endpoint
**Prezalumab (AMG557)** **(MEDI5872)** **Anti-B7RP1**	NCT02334306 Phase IIa [363]	AECG criteria and ESSDAI ≥ 5 SSa and/or SSb+ FR+, cryoglobulinemia or hypergammaglobulinemia	16	16	50.7 ± 13	?	?	ΔESSDAI at 99d	Not met	ESSPRI =	ESSPRI =	ESSDAI =
**Lulizumab (BMS-931699)** **Anti-CD28**	NCT02843659 Phase II [351]	2016 ACR/EULAR criteria SSa and/or SSb+ ESSDAI ≥ 5 USW > 0.01 mL/min	5+6	7	51.2 ± 11.41	?	?	ΔESSDAI at 12w	Not published	Secondary endpoint	Secondary endpoint	Primary endpoint

**Table 10:** T-cell targeted drugs in pSS: therapies preventing autoantigen presentation.

DRUG	TRIAL (Reference)	Inclusion Criteria	Number of Subjects		Age (Years)	Disease Duration (Years)	Mean ESSDAI	Primary Outcome	Results	Effects (Statistically Significant)		
			Drg	Ctrl						Sicca Syndrome	Fibro-Like	Systemic
**Petesicatib** **RO5459072** **Cathepsin S** **Inhibitor**	NCT02701985 Phase IIa [364]	AECG criteria SSa and/or SSb+ ESSDAI ≥ 5 ESSPRI ≥ 5 USW > 0.0 mL/min Oral D-VAS ≥ 5/10	38	37	52.2 ± 12.5	?	?	ΔESSDAI ≥ 3 at 12w	Not met	ESSPRI =	ESSPRI = SF36 =	ESSDAI =
**Efalizumab** **Anti-LFA-1**	NCT00344448 Phase II [365]	AECG criteria SSa and/or SSb+	6	3	53 ± 11.2	?	?	Protocol-specified composite score at 12w	Early termination due to serious side effect in other trial			

**Table 11:** Anti-cytokine targeted drugs in pSS.

DRUG	TRIAL	Inclusion Criteria	Number of Subjects		Age (Years)	Disease Duration (Years)	Mean ESSDAI	Primary Outcome	Results	Effects (Statistically Significant)		
			Drg	Ctrl						Sicca Syndrome	Fibro-Like	Systemic
**Anakinra** **IL1R antagonist protein**	NCT00683345 Phase II [333]	AECG criteria 18–80 years Western European descent No depression or comorbidity	13	13	55 (36–80)	5 (1–17)	?	Group-wise comparison of the fatigue scores at 4w	not met	na	F-VAS ⇩	na
**Tocilizumab** **Anti-IL-6R**	NCT01782235 Phase I/II ETAP [366]	AECG criteria ESSDAI ≥ 5	55	55	50.9 (26–76)	?	11.5 (5–25)	ΔESSDAI ≥ 3 at 12W without new item without ⇧ ≥1/10 PGA	not met	ESSPRI = Schirmer =	ESSPRI =	ESSDAI = Articular ⇩
**Infliximab** **Anti-TNF**	Phase III TRIPSS [367]	AECG criteria 2/3 D-VAS, F-VAS, P-VAS ≥ 5/10	54	49	54.4 ± 10.4	4.0 ± 5.5	na	⇧ 30% in 2/3 D-VAS, F-VAS, P-VAS at 10–22w	not met	SWS = Schirmer =	SF-36 =	SJC = TJC =
**Etanercept** **TNFR-Ig fusion protein**	NCT00001954 Phase II [368]	1986 and AECG criteria Elevated ESR or IgG levels	14	14	55.5 (46, 59)	?	na	⇧ 20% in 2/3 pSS domains (protocol-specified)	not met	D-VAS = Schirmer = VB = SWS =	na	na
**Ustekinumab** **Anti-** **IL-12/IL-23** **(p40 subunit)**	NCT04093531 Phase I [341]	2016 ACR/EULAR criteria	15	-	?	?	?	ΔESSDAI at 24W	Estimated Study Completion on December 2021	na	Secondary endpoint	Primary endpoint
**GSK2618960** **anti–IL-7Rα**	NCT03239600 Phase II [369]	AECG criteria SWS >0.1 mL/min ⇧ Ig or FR+ or ANA ≥ 1:320 D-VAS ≥ 5/10 or Schirmer < 10 mm	0		-	-	-	SAEs at 27w	Withdraw The study is stopped for Portfolio prioritization			

**Table 12:** Miscellaneous targeted drugs in pSS.

DRUG	TRIAL (Reference)	Inclusion Criteria	Number of Subjects		Age (years)	Disease Duration (Years)	Mean ESSDAI	Primary Outcome	Results	Effects (Statistically Significant)		
			Drg	Ctrl						Sicca Syndrome	Fibro-Like	Systemic
**Daxdilimab** **VIB7734** **Anti-ILT7**	NCT03817424 Phase I [370]	Unspecified	?	?	**?**	**?**	SAEs at 169d AESIs at 169d	June 2020	na	na	na	na
**Filgotinib** **Jak1 inhibitor**	NCT03100942 Phase II [342]	AECG criteria SSa and/or SSb+ ESSDAI ≥ 4	38	37	52.2 ± 10.54	?	10.2 ± 6.23	Protocol-Specified Response Criteria at 12w	not met	ESSPRI =	ESSPRI =	ESSDAI =
**Lanraplenib** **(GS-9876)** **SIK inhibitor**	NCT03100942 Phase II [342]	AECG criteria SSa and/or SSb+ ESSDAI ≥ 4	38	37	56.2 ± 9.72	?	10.5 ± 4.89	Protocol-Specified Response Criteria at 12w	not met	ESSPRI =	ESSPRI =	ESSDAI =
**RSLV-132** **RNase1-Fc fusion protein**	NCT03247686 Phase II [371]	AECG criteria SSA+ Interferon signature	22	8	?	?	?	Interferon gene expression at day99	Not published	ESSPRI =	mPRO-F ⇧ DSST ⇩ FACIT-F = ESSPRI =	ESSDAI =
**Low-dose** **IL-2** **T-reg induction**	NCT01988506 Phase II Transreg [372]	pSS diagnosis	84-132		?	?	?	T-reg percentage	Estimated Study Completion on February 2022	na	na	na

## Abbreviations

ACA	Anti-centromere antibodies
ACPA	Anti-citrullinated protein antibodies
ACR	American College of Rheumatology
AECG	American European Consensus Group
AH	Autoimmune Hepatitis
ANA	Antinuclear antibodies
anti-M3R	Anti-muscarinic receptor 3
APRIL	A proliferation-inducing ligand
ASAP	“Abatacept Sjögren Active Patients” study
AZA	Azathioprine
BAFF	B cell Activating Factor
BCR	B cell receptor
BUT	Break-up Time
CCP	Cyclic Citrullinated Peptide
circRNA	Circular RNA
ciRNAs	Intronic circRNAs
ClinESSDAI	Clinical ESSDAI variant
CPK	Creatine phosphokinase
CRISP-3	Cysteine-Rich Secretory Protein 3 ()
CT-scan	Computerized tomography
CyA	Ciclosporin A
DAMPS	Danger-associated molecular patterns
DAP-kinase	Pro-apoptotic death associated protein kinase
DHEA	Dehydroepiandrosterone
DHT	Dihydrotestosterone
DLBCL	Diffuse large B cell lymphoma
DMARD	Disease Modifying Anti-Rheumatic Drug
DNMTs	DNA methyltransferases
DREAM	“Dry Eye Assessment and Management” study
EBV	Epstein-Barr virus
ecircRNAs	Exonic circRNAs
EIciRNAs	Exon-intron circRNAs
ELISA	Enzyme-linked immunosorbent assay
ENT	Ear-Nose-Throat
ESSDAI	EULAR Sjögren’s syndrome disease activity index
ESSPRI	EULAR Sjögren’s Syndrome Patient Reported Index
EULAR	European League Against Rheumatism
FASl	Fas ligand
FDC	Follicular dendritic cells
GCs	Germinal centres
HCQ	Hydroxychloroquine
HCV	Hepatitis C virus
HLH	Hemophagocytic lymphohistiocytosis
HTLV1	Human T-lymphotropic virus type I
ICAM-1	InterCellular Adhesion Molecule 1
IF	Immunofluorescence
IFN	Interferon
IgG,A,M	Immunoglobulin G, A and M
IL-	Interleukin
ILD	Interstitial lung disease(s)
IRF	Interferon Regulatory Factor
IV	Intravenous therapy
IVIG	Intravenous Immunoglobulin
KCS	Keratoconjunctivitis sicca
LEMA	Myoepithelial sialadenitis
LESA	lymphoepithelial sialadenitis
LIP	Lymphocytic interstitial pneumonitis
LMP1	Latent membrane protein 1
lncRNA	Long non-coding RNAs
LPR	Laryngopharyngeal reflux
LSG	Labial SG
MALT	mucosa-associated lymphoid tissue
MHC	Major histocompatibility genes
MMF	Mycophenolate mofetil
MMP	Matrix metalloproteinases
MPGN	Mesangioproliferative glomerulonephritis
MRI	Magnetic Resonance Imaging
MS	Multiple Sclerosis
MSGB	Minor salivary gland biopsy
MTX	Methotrexate
NAC	N-acetylcystein
NFkB	Nuclear factor kappa-light-chain-enhancer of activated B cells
NHL	Non-Hodgkin’s lymphoma
NICE	National Institute for Health and Care Excellence
NMOSD	Neuromyelitis optica spectrum disorder
NOD	Non-obese diabetic
NSAID	Nonsteroidal anti-inflammatory drugs
NSIP	Nonspecific interstitial pneumonia
OMERACT	Outcome Measures in Rheumatology group
OSDI	Ocular Surface Disease Index
OSS	Ocular Staining Score
PAMPs	Pathogen-associated molecular patterns
PBC	Primary Biliary Cirrhosis
PDC	Plasmacytoid dendritic cells
PDL1	Programmed death ligand 1
PET scan	Positron emission tomography
PGA	Patient Global Assessment
PIP	Prolactin inducible protein
PO	per os
PSP	Parotid secretory protein
pSS	Primary Sjögren’s Syndrome
pSS-ILD	pSS-related interstitial lung disease
q6h, q8h	Every 6 h, every 8 h
RA	Rheumatoid Arthritis
RCT	Randomized controlled trial
RF	Rheumatoid Factor
RTA	Renal tubular acidosis
RTX	Rituximab
RX1	Runt-related transcription factor
SAM	Methyl donor S-adenosylmethionine
SAP	Sicca Asthenia Polyalgia
SF-36	Short Form 36 health survey
SG	Salivary Gland
SGS	Salivary glands scintigraphy
SGUS	Salivary glands ultrasound
SICCA	Sjögren’s International Collaborative Clinical Alliance
SLE	Systemic lupus erythematosus
SNP	Single nucleotide polymorphism
SP-1	Salivary protein 1
SSDDI	Sjögren’s Syndrome Disease Damage Index
SSDI	Sjögren’s Syndrome Damage Index
sSS	Secondary Sjögren’s Syndrome
SWSF	Stimulated Whole Salivary Flow rate
TACI	Transmembrane Activator and CAML Interactor
TEARS	“Tolerance and efficacy of rituximab in primary Sjögren syndrome” trial
Tfh	Follicular helper T cells
TLRs	Toll Like Receptors
TNF-α	Tumour necrosis factor-α
TPHA	Treponema Pallidum Hemagglutinations Assay
TRACTISS	“TRial of Anti-B-Cell Therapy In patients with primary Sjögren’s Syndrome” trial
TSH	Thyroid-stimulating hormone
TTP	Thrombotic Thrombocytopenic Purpura
UCLH	University College London Hospitals
UIP	Usual interstitial pneumonia
UWSF	Unstimulated Whole Saliva Flow rate
VAS	Visual analogue scales
VDRL	Venereal Disease Research Laboratory
